# Phase Diversity Electro-optic Sampling: A new approach to single-shot terahertz waveform recording

**DOI:** 10.1038/s41377-021-00696-2

**Published:** 2022-01-10

**Authors:** Eléonore Roussel, Christophe Szwaj, Clément Evain, Bernd Steffen, Christopher Gerth, Bahram Jalali, Serge Bielawski

**Affiliations:** 1 Univ. Lille, CNRS, UMR 8523 - PhLAM - Physique des Lasers, Atomes et Molécules, Centre d’Étude Recherches et Applications (CERLA), F-59000 Lille, France; 2 DESY (Deutsches Elektronen-Synchrotron), Notkestr. 85, D-22607 Hamburg, Germany; 3 Electrical and Computer Engineering Department, University of California, Los Angeles, 420 Westwood Plaza, 90095 Los Angeles, CA USA

**Keywords:** Ultrafast photonics, Free-electron lasers, Terahertz optics

## Abstract

Recording electric field evolution in single-shot with THz bandwidth is needed in science including spectroscopy, plasmas, biology, chemistry, Free-Electron Lasers, accelerators, and material inspection. However, the potential application range depends on the possibility to achieve sub-picosecond resolution over a long time window, which is a largely open problem for single-shot techniques. To solve this problem, we present a new conceptual approach for the so-called spectral decoding technique, where a chirped laser pulse interacts with a THz signal in a Pockels crystal, and is analyzed using a grating optical spectrum analyzer. By borrowing mathematical concepts from photonic time stretch theory and radio-frequency communication, we deduce a novel dual-output electro-optic sampling system, for which the input THz signal can be numerically retrieved—with unprecedented resolution—using the so-called phase diversity technique. We show numerically and experimentally that this approach enables the recording of THz waveforms in single-shot over much longer durations and/or higher bandwidth than previous spectral decoding techniques. We present and test the proposed DEOS (Diversity Electro-Optic Sampling) design for recording 1.5 THz bandwidth THz pulses, over 20 ps duration, in single-shot. Then we demonstrate the potential of DEOS in accelerator physics by recording, in two successive shots, the shape of 200 fs RMS relativistic electron bunches at European X-FEL, over 10 ps recording windows. The designs presented here can be used directly for accelerator diagnostics, characterization of THz sources, and single-shot Time-Domain Spectroscopy.

## Abstract (web summary)

We demonstrate a new strategy for recording THz electric signals with long duration and high bandwidth. It uses an original combination of Terahertz electro-optic detection techniques, with diversity concepts borrowed from wireless communications and time-stretch.

## Graphical abstract

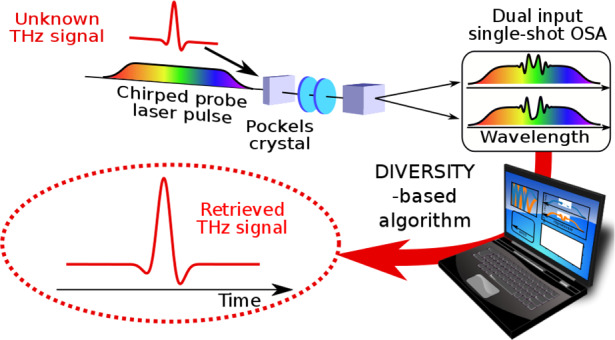

## Introduction

Recording the complete electric field of light in single-shot (including its envelope and carrier) is considered one of the “holy grails” of terahertz science. This type of detection is largely needed for investigating and mastering novel terahertz sources, as ultrashort pulse quantum cascade lasers^1^, ultra-wide bandwidth laser-plasma-based terahertz sources^2^, and terahertz Free-Electron-Lasers^3^. Such tools are also crucial for mastering novel “extreme photonic infrastuctures”, such as X-ray Free-Electron Lasers^4^, and in the very active field of Laser-Plasma Acceleration (LPA)^5^, which has the ambition to replace large accelerators facilities by table-top installations. Single-shot terahertz recorders are also needed in applications, such as spectroscopy, using high power THz sources. In this case, the low repetition rate of the sources makes traditional methods of time-domain spectroscopy (TDS) (based on scanning the delay between a probe laser and the THz signal under interest) largely impractical.

**Fig. 1:**
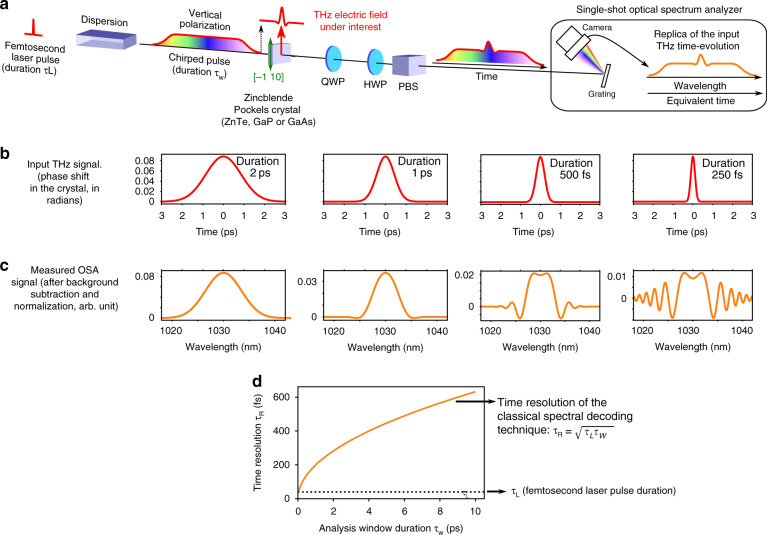
Principle and limitations of classical single-shot THz waveform recorders using time-to-spectrum conversion (also known as spectral decoding). **a** Principle. The electric field pulse shape modulates the birefringence of a Pockels electro-optic crystal. A probe chirped laser pulse (with stretched duration *τ*_*w*_) is then intensity-modulated in single shot after passing the Pockels crystal, the quarter and half-wave plates (QWP and HWP) and the polarizing beam-splitter (PBS). Because of the laser chirp, the input temporal shape is expected to be “replicated” in the laser spectrum recorded by the grating OSA. **b**, **c** Fundamental time-resolution limitation of the method (numerical simulation assuming a perfect crystal with infinite bandwidth). The method is unreliable (i.e., strong deformations occur) when the input THz pulse is shorter than *τ*_*R*_ = 0.7 ps, although a 39 fs femtosecond laser is used. **d** Resolution limitation of the classical method (orange, from Eq. 1). An objective of DEOS is to remove this limitation, and obtain a resolution that does no more degrades when the duration of the analysis window *τ*_*w*_ is increased. The probe laser compressed duration *τ*_*L*_ (black line) is given for reference. Laser parameters: 1030 nm wavelength and 40 nm FWHM bandwidth (i.e., *τ*_*L*_ = 39 fs compressed laser pulse duration). *τ*_*w*_ = 10 ps FWHM. See Table 1 for crystal orientations, and “Materials and methods” for details).

However recording a complete terahertz wave in single-shot is a largely open problem, when a large bandwidth and long record duration are simultaneously needed. Promising strategies consist in extending the so-called electro-optic sampling technique^6^ to the single-shot case. A popular strategy consists of imprinting the electric field evolution onto a chirped laser pulse^7–9^, and recording the optical spectrum using a grating-based optical spectrum analyzer (OSA), as displayed in Fig. 1a. This method is now usually known as *spectral encoding* or *spectral decoding*.

The initial idea^7^ has been based on the assumption that—because the THz signal is imprinted onto the time-evolution of a chirped laser pulse—we can expect the THz signal shape to appear, with a good fidelity, in the output optical spectrum. However, it was shown that this idea works only if the needed temporal resolution is larger than^8^:

1 $${\tau }_{R}=\sqrt{{\tau }_{w}\times {\tau }_{L}}$$

where *τ*_*w*_ is the duration of the chirped pulse on the crystal, and *τ*_*L*_ is the Fourier-transform limit duration (i.e., the pulse duration that may be reached when fully compressed).

It is important to note that this limitation is much more drastic than a simple “blurring effect”. When time evolutions faster than *τ*_*R*_ are present in the THz signal, the output signal is typically a very deformed version of the input signal, as displayed in Fig. 1c. Moreover, the limitation set by Eq. 1 dramatically worsens when the desired analysis window duration increases (see Fig. 1d). As a concrete example, in a situation where a recording duration of 10 ps is required, and a 100 fs laser is available, the resolution *τ*_*R*_ would be limited to 1 ps. More generally this limitation implies that the resolution *τ*_*R*_ will be much larger than the initial pulse duration *τ*_*L*_, this issue being worse as the desired analysis window *τ*_*w*_ increases.

This major issue has been the subject of active investigations during the last two decades. Deconvolution algorithms have only led to limited improvements^10,11^, and most research switched to alternate hardware strategies, such as encoding the information onto the transverse or angular direction^12–16^, or combining spectrally decoded electro-optic sampling with advanced laser pulse characterization techniques (such as FROG)^17,18^. This led to improvements of the temporal resolution. However, these complex designs introduce new trade-offs between sensitivity, maximum recording duration, temporal resolution, and repetition rate.

Here we show that it is possible to considerably increase the temporal resolution of *spectral decoding* measurement systems by using a new approach that we call Diversity Electro-Optic Sampling (DEOS). This modification allows us to “break” the previous fundamental barrier displayed by Eq. 1, and reach sub-picosecond resolution without fundamental limitation on the window of analysis. Key to our approach is a completely new conceptual approach of spectrally decoded electro-optic sampling, which has its roots in the theory of the *photonic time-stretch analog-to-digital converter*^19,20^ where a similar problem called dispersion penalty was identified and solved ^21^. As we will see, this point of view provides a way to remove the temporal resolution limit by using a technique known as *phase diversity*^21^, and which makes use of a dual-output electro-optic sampling system. Note that the name phase diversity comes from its use in time stretch systems and pays homage to antenna diversity, a technique that eliminates the transmission fading produced by multipath interference, by using several transmission channels (i.e., several antenna).

We describe the DEOS technique and validate it on two very different experimental applications. First, we show how to achieve phase diversity utilizing polarization modulation in an electro-optic crystal and demonstrate single-shot recording of THz pulses in a table-top environment. This provides an experimental validation of the method, as well as the building blocks of a novel “single-shot TDS” system. Second, we demonstrate the DEOS approach in an electron accelerator, by probing the Coulomb-field of electron bunches at megahertz repetition rates, at the European X-ray Free-Electron Laser^4^ (EuXFEL). This experiment achieved sub-200 fs resolution over a time window in the 10 ps range.

## Results

### DEOS single-shot recorder: experimental technique and novel theoretical framework

**Fig. 2:**
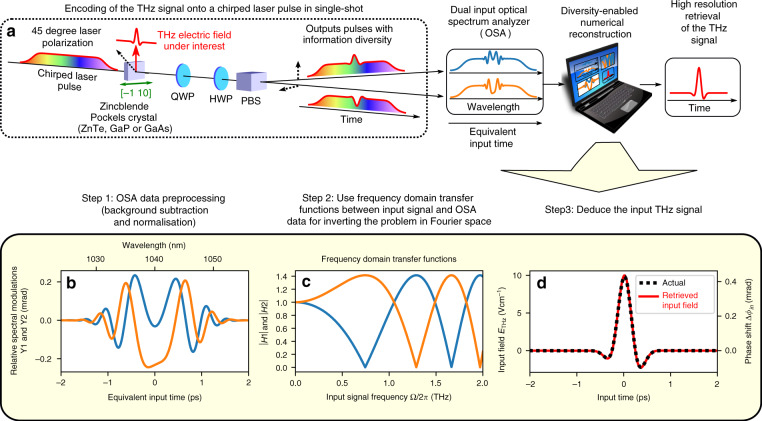
Principle of Phase Diversity Electro-optic Sampling, DEOS, for single-shot recording of THz electric fields. **a** Experimental design: The input THz field evolution modulates a chirped laser pulse, and the DEOS design provides two outputs that contain different information. Using this two-output design (see Table 1 for details), the recorded information is sufficient for removing the problem, i.e., retrieving the input signal with high resolution. **b**−**d** Main steps of our reconstruction method (numerical simulation). **b** Raw electro-optic sampling (EO) signals (optical spectra after background subtraction and normalization). **c** Transfer functions *H*_1_ and *H*_2_ corresponding to the two polarization outputs showing the phase diversity operation. **d** Input signal retrieved from the recorded OSA signals [displayed in (**b**)] and the transfer functions *H*_1,2_(Ω) [displayed in (**c**)], using the MRC algorithm (Eq. 10). Laser parameters close to those of the European XFEL experiment presented hereafter (1040 nm wavelength and 40 nm FWHM bandwidth, *τ*_*w*_ = 5 ps FWHM).

Our experimental method is displayed in Fig. 2a. DEOS is based on chirped pulse electro-optic sampling, with a readout that uses a grating spectrometer^7,8,22–27^, but with crucial modifications that remove the temporal resolution limitation. The electric field under test *E*(*t*) is imprinted in single-shot onto the intensity and phase of a chirped laser pulse. If we assume a linear chirp, the optical angular frequency at the spectrometer *ω*_opt_ is related to the input time *t* by:

2 $$t=-\frac{{\omega }_{{\rm{opt}}}-{\omega }_{{\rm{opt}}}^{{\rm{center}}}}{C}$$

where *C* = ∂*ω*_opt_/∂*t* is the laser chirp rate.

A key innovation in DEOS is how to achieve phase diversity in polarization-based electro-optic modulation. To do so, we record the optical spectra in single-shot at the two polarizer outputs, and the two optical spectra provide the input data of our retrieval algorithm. The optical spectra are first passed through a pre-processing pipeline that consists of subtraction, amplitude scaling, and optical wavelength to time conversion (see “Materials and methods”). These signals *Y*_1_(*t*) and *Y*_2_(*t*) will be named the EO (electro-optic) signals in what follows. Examples of EO signals are displayed in Fig. 2b. A key feature of the DEOS method that creates diversity is the special arrangement of the crystal, waveplates, and polarizations (see Table 1), which leads to *diversity in frequency response* on the two output channels. This diversity will enable a retrieval of the input terahertz signal using the maximum ratio combining^21,28^.

**Table 1:** Differences in crystal and waveplates orientations between classic spectrally decoded EO sampling (Fig. 1) and the new phase diversity (DEOS) system (Fig. 2).

	Classic spectrally decoded EO sampling	DEOS
Pockels crystal	[-110] axis parallel to THz field	[-110] perpendicular to THz field
Input laser polarization	at 0 or 90 degrees wrt THz field	at 45 degrees wrt THz field
QWP (*)	one axis at 45 degrees wrt [-110]	one axis along [-110]
HWP (*)	optional	at 22.5 degrees wrt [-110]
Transfer functions *H*_1_ and *H*_2_		

For the classical system, we indicate the most commonly used crystal orientation, which corresponds to maximal Pockels effect. (*): For classic spectrally decoded EO sampling, several quarter wave plates (QWP) and half wave plates (HWP) configurations are possible and we indicate here the angles for the so-called balanced detection. Note that for DEOS, the orientations are chosen in order to obtain information phase diversity at the two outputs, i.e., transfer functions with interleaved zeros (see also Fig. 2c). Ω is the input THz signal angular frequency, and *C* is the laser chirp rate (see text and Eq. 2).

At first glance, it may not be obvious how the two output channels exiting the polarizing beam-splitter achieve phase diversity. While the two outputs may appear to carry the same information, as displayed in Table 1, the *phase* modulations will be different. This *phase diversity* phenomenon^21^ will thus lead to the two OSA channels to have complementary null behavior (see Fig. 2). We will see below that the combined information in *Y*_1_(*t*) and *Y*_2_(*t*) will eliminate the nulls in the frequency response and hence overcome the fundamental bandwidth limitation.

### Theoretical framework for Phase Diversity

In this section we derive the relation between the input *E*(*t*) and the outputs *Y*_1_(*t*) and *Y*_2_(*t*) measured by the single-shot grating-based OSA. After a relatively non-trivial calculation (see Supplementary Material, Sections V, VI), it can be shown that, in Fourier domain, the input and outputs are related by remarkably simple analytic expressions involving *transfer functions* *H*_1_ and *H*_2_:

3 $${\tilde{Y}}_{1}({{\Omega }})\approx {H}_{1}({{\Omega }})\tilde{{{\Delta }}{\phi }_{{\rm{in}}}}({{\Omega }})$$

4 $${\tilde{Y}}_{2}({{\Omega }})\approx {H}_{2}({{\Omega }})\tilde{{{\Delta }}{\phi }_{{\rm{in}}}}({{\Omega }})$$

where the tilde denotes the Fourier transform, and Ω is the terahertz angular frequency at the input. Δ*ϕ*_in_(*t*) is the optical phase shift induced by the THz field *E*(*t*) in the crystal birefringence:

5 $${{\Delta }}{\phi }_{{\rm{in}}}(t)=\beta E(t)$$

6 $$\,{{\mbox{with}}}\,\ \beta =\frac{\pi d}{\lambda }{n}_{0}^{3}{r}_{41}$$

where *n*_0_ is the refractive index at vanishing electric field, *d* is the thickness of the crystal and *r*_41_ is the electro-optic coefficient. *λ* is the laser wavelength in vacuum and *E*(*t*) the electric field inside the crystal. The transfer function approach is valid for small values of Δ*ϕ*_in_(*t*).

For the specific DEOS design described in Fig. 2a and Table 1, the corresponding transfer functions can be written as (see Supplementary Material, Section VI):

7 $${H}_{1}({{\Omega }})=\sqrt{2}\cos \left(B{{{\Omega }}}^{2}+\frac{\pi }{4}\right)$$

8 $${H}_{2}({{\Omega }})=-\sqrt{2}\cos \left(B{{{\Omega }}}^{2}-\frac{\pi }{4}\right)$$

where $$B=\frac{1}{2C}$$.

As a first observation, the transfer functions *H*_1_(Ω) and *H*_2_(Ω) present nulls at specific frequencies (Fig. 2c). These nulls limit the temporal resolution and the loss of signal at or near the null frequencies cannot be reversed by deconvolution (i.e., to retrieve the input field *E*(*t*) from the recorded data). This theoretical result on the existence of nulls is consistent with previous experimental observations^29,30^, and ours, as we will see below. Conceptually, these nulls correspond to the dispersion penalty phenomenon observed in the photonic time-stretch digitizer^21^, as shown in Supplementary Material, Sections VB and VIA.

With the deep insight gained from the theoretical analysis above, we can recover the input signal Δ*ϕ*_in_(*t*) and *E*(*t*) by exploiting the information contained in both output channels because *H*_1_(Ω) and *H*_2_(Ω) are complementary (diverse). In this respect, it is important to note that the relative positions of the zeros (nulls) depend on the crystal orientations. For instance, the polarization orientations used traditionally ([-110] axis parallel to the terahertz field—see Table 1 and in Fig. 1) would not work, as this would lead to nulls of *H*_1,2_ that occur at the same frequencies (see Supplementary Fig. S4). In addition, the polarization arrangement considered in this article—for which the zeros are complementary—is only one of many possible choices which are compatible with the following retrieval algorithm.

### Maximal ratio combining (MRC) algorithm for signal reconstruction

As the zeros (nulls) of the transfer functions are interleaved, we can retrieve the unknown THz electric field from the recorded data. The mathematical problem is well-posed, and even overdetermined, i.e., one has the freedom to chose either *H*_1_ or *H*_2_ for inverting the problem, except at the nulls for which both channels must be used. Several advanced methods exist for the reconstruction process. Here we use the so-called MRC technique^21,28^, which is designed for optimizing the signal-to-noise ratio (SNR). The input signal can be retrieved from the measurements *Y*_1,2_ as^21^:

9 $${\tilde{E}}_{{\rm{in}}}^{{\rm{retr}}}({{\Omega }})=\frac{1}{\beta }\tilde{{{\Delta }}{\phi }_{{\rm{in}}}^{{\rm{retr}}}}({{\Omega }})$$

10 $$\,{{\mbox{with}}}\,\ \tilde{{{\Delta }}{\phi }_{{\rm{in}}}^{{\rm{retr}}}}({{\Omega }})=\frac{{H}_{1}({{\Omega }}){\tilde{Y}}_{1}({{\Omega }})+{H}_{2}({{\Omega }}){\tilde{Y}}_{2}({{\Omega }})}{{H}_{1}^{2}({{\Omega }})+{H}_{2}^{2}({{\Omega }})}$$

where ${\tilde{E}}_{{\rm{in}}}^{{\rm{retr}}}({{\Omega }})$ and $\tilde{{{\Delta }}{\phi }_{{\rm{in}}}^{{\rm{retr}}}}({{\Omega }})$ are the retrieved input electric field and crystal phase modulation, expressed in Fourier space. The input signal ${E}_{{\rm{in}}}^{{\rm{retr}}}(t)$ (or equivalently ${{\Delta }}{\phi }_{{\rm{in}}}^{{\rm{retr}}}(t)$) is then obtained by performing an inverse Fourier transform.

Note that the theoretical transfer function of DEOS, defined from the input THz-induced crystal phase-shift ${{\Delta }}\tilde{\phi }({{\Omega }})$ to the reconstructed signal ${{\Delta }}{\tilde{\phi }}_{{\rm{in}}}^{{\rm{retr}}}({{\Omega }})$ is—by design—perfectly flat. This can be easily verified by injecting the definitions of the single channel transfer functions (Eqs. 3, 4) in the MRC reconstruction Eq. 10. One easily finds:

11 $${{\Delta }}{\tilde{\phi }}_{{\rm{in}}}^{{\rm{retr}}}({{\Omega }})={H}_{{\rm{MRC}}}({{\Omega }}){{\Delta }}{\tilde{\phi }}_{{\rm{in}}}({{\Omega }})$$

12 $$\,{{\mbox{with}}}\,\ {H}_{{\rm{MRC}}}({{\Omega }})=1$$

or, after an inverse Fourier transform:

13 $${{\Delta }}{\phi }_{{\rm{in}}}^{{\rm{retr}}}(t)={{\Delta }}{\phi }_{{\rm{in}}}(t)$$

This implies that any sharp input temporal feature Δ*ϕ*_in_(*t*) with bandwidth Ω_max_ should now be retrieved without distortion, provided the corresponding input data ${\tilde{Y}}_{1}({{\Omega }})$ and *Y*_2_(Ω) can be obtained with reasonable SNR, up to Ω_max_.

We tested this reconstruction method numerically for various parameters, and found that it is possible to retrieve the input pulse for arbitrarily long input chirped pulses (i.e., for any duration of the analysis window), down to terahertz pulse durations of the order of the Fourier-limited pulse duration (see “Discussion”, Suplementary Material, Section VIII). An example of retrieved input pulse is presented in Fig. 2d.

### Experimental demonstration: recording free-propagating terahertz pulses with large time-bandwidth products

**Fig. 3:**
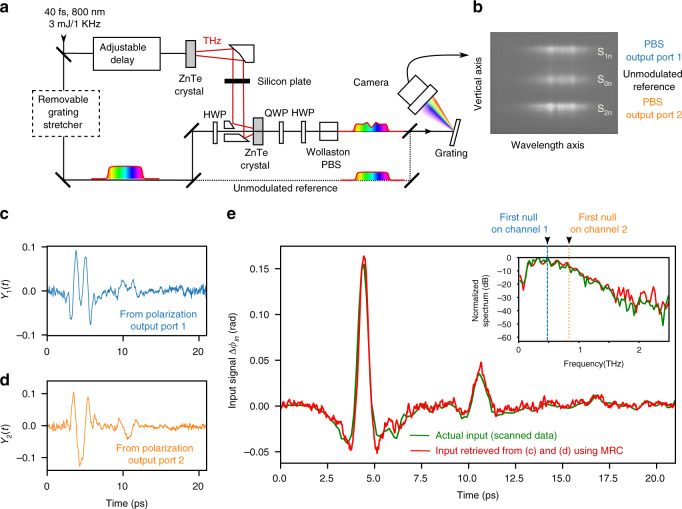
Single-shot recording of free-propagating terahertz pulses over a window of the order of 20 ps. **a** DEOS experimental setup. ZnTe: 1 mm-thick, 110-cut Zinc Telluride crystal, HWP: Half-wave plates, QWP: Quarter-wave plate, PBS: Wollaston polarizing cube beam-splitter. The beams emerging from the PBS are in the plane perpendicular to the figure. **b** Raw camera image containing the single-shot spectra of the two polarization outputs *S*_1,2*n*_, and the unmodulated laser spectrum *S*_0*n*_. **c**, **d** EO signals on two polarizations channels (after background subtraction and normalization, see “Materials and methods”. **e** Single-shot input signal retrieved from (**c**) and (**d**) using the DEOS phase-diversity-based algorithm (red). Green trace: actual input, obtained using scanned electro-optic sampling. Inset: Fourier spectra of the two terahertz signals. Note that the classical (i.e., single channel) spectral decoding method would just provide the deformed signals (**c**) or (**d**), depending on the channel used. More generally, a classical single-channel method would provide an input signal with good fidelity only if its bandwidth is small compared to the location of the first transfer function zero (dashed lines in the inset of (**e**)), or if the signal bandwidth is within one of the lobes in the frequency response in Fig. 2b.

In order to test the DEOS method experimentally, we first constructed the setup displayed in Fig. 3a. Terahertz pulses are produced by optical rectification of 800 nm millijoule-range laser pulses in a Zinc Telluride (ZnTe) crystal. These terahertz pulses are then analyzed by an EO sampling setup based on our design.

For test purposes, we also designed the setup so that it is possible to skip or operate the grating stretcher, without changing the setup alignment. We can thus analyze the terahertz pulses using either single-shot “realtime” EO sampling (i.e., using chirped pulses) or using the traditional “equivalent-time” EO sampling by scanning the delay between the femtosecond laser pulses and the terahertz signal. We will thus test our single-shot method by comparing the DEOS results to the corresponding scanned EO signals.

A typical result is presented in Fig. 3e, using a time window of 20 ps. The reconstructed EO signal and the reference EO signal (i.e., obtained using scanned electro-optic sampling) are found to be extremely similar in shape. The reconstruction is even able to reproduce fine details, as the small oscillations between 5 and 20 ps, which are due to the water vapor absorption in air (free-induction decay), and multiple reflections on the Silicon plate. For comparison, the EO signal shapes *Y*_1,2_ before reconstruction (i.e., corresponding to the previous state-of-art) are very different from the input, as can be seen in Fig. 3c, d.

These results confirm that the DEOS method now enables investigations of terahertz sources, as well as TDS to be achieved in single-shot, with simultaneously high temporal and spectral resolution. More precisely the time resolution and bandwidth limit of the DEOS method appear—as theoretically expected—similar to classical (and non-single-shot) “scanned” electro-optic sampling.

### Note: determination of the reconstruction parameter

As this type of reconstruction requires the knowledge of the transfer functions *H*_1,2_, it is important to find a practically convenient approach for determining the parameter *B*. We remarked that *B* can be determined in a very simple way, by analyzing the recorded data corresponding to the unknown signal. From the reconstructed signal ${{\Delta }}{\phi }_{{\rm{in}}}^{{\rm{retr}}}(t)$, we can simulate the corresponding DEOS signals ${\tilde{Y}}_{1}^{{\rm{retr}}}({{\Omega }})$ and ${\tilde{Y}}_{2}^{{\rm{retr}}}({{\Omega }})$:

14 $${\tilde{Y}}_{1}^{{\rm{retr}}}({{\Omega }})={H}_{1}({{\Omega }})\tilde{{{\Delta }}{\phi }_{{\rm{in}}}^{{\rm{retr}}}}({{\Omega }})$$

15 $${\tilde{Y}}_{2}^{{\rm{retr}}}({{\Omega }})={H}_{2}({{\Omega }})\tilde{{{\Delta }}{\phi }_{{\rm{in}}}^{{\rm{retr}}}}({{\Omega }})$$

where *H*_1,2_, ${\tilde{Y}}_{1,2}^{{\rm{retr}}}$ and $\tilde{{{\Delta }}{\phi }_{in}^{{\rm{retr}}}}({{\Omega }})$ depend on *B*.

Then we can perform a least-square fit of $\tilde{{Y}_{1}^{{\rm{retr}}}}$ and $\tilde{{Y}_{2}^{{\rm{retr}}}}$ on $\tilde{{Y}_{1}}$ and $\tilde{{Y}_{2}}$, using *B* as a free parameter. Here, we perform a classical least-square fit using the following definition for the reconstruction error *ϵ*:

16 $${\epsilon }^{2}=\int\nolimits_{-\infty }^{+\infty }{\rm{d}}{{\Omega }}\left({\left|{\tilde{Y}}_{1}-{\tilde{Y}}_{1}^{{\rm{retr}}}\right|}^{2}+{\left|{\tilde{Y}}_{2}-{\tilde{Y}}_{2}^{{\rm{retr}}}\right|}^{2}\right)$$

**Fig. 4:**
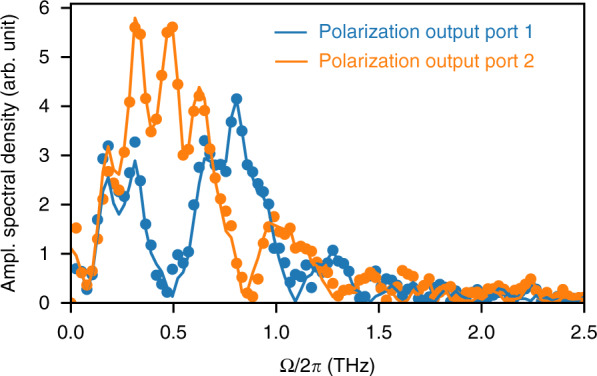
Fit providing the reconstruction parameter *B* from a single-shot recording. Dots: Fourier spectra $| {\tilde{Y}}_{1,2}({{\Omega }})|$ of experimental data before reconstruction. Lines: spectra $| {\tilde{Y}}_{1,2}^{{\rm{retr}}}({{\Omega }})|$ computed from the retrieved input. ${\tilde{Y}}_{1,2}^{{\rm{retr}}}({{\Omega }})$ are fitted on ${\tilde{Y}}_{1,2}({{\Omega }})$ using *B* as a free parameter. Note the presence of interleaved zeros, which play a key role in the retrieval approach. Same data and color codes as for Fig. 3c, d.

Besides its use for checking the fit quality, it is interesting to examine the reconstructed ${\tilde{Y}}_{1,2}^{{\rm{retr}}}$ and raw ${\tilde{Y}}_{1,2}$ EO signals (Fig. 4). Those curves clearly display the nulls that stem from the zeros of the transfer function *H*_1,2_ expressed in Eqs. (7, 8).

Remarkably, this result shows that the determination of the fit parameter *B* does not require specific experiments to be performed. The fit can be made using any unknown input signal—including the signal to be analyzed—provided its bandwidth is sufficient.

### Application to measurements of relativistic electron bunch shapes at the European X-ray free-electron laser

The high resolution retrieval based on phase diversity is expected to find immediate applications in single-pass Free-Electron Lasers (FELs), such as EuXFEL^4^. FELs are based on self-amplification of stimulated radiation driven by ultra-short relativistic electron bunches with high peak current propagating in a periodic magnetic field (undulators). They provide femtosecond photon pulses with energies in the millijoule range, and wavelengths ranging from the ultraviolet to hard X-rays depending on the facilities. The emitted photon pulses depend directly on the properties of the electron bunches. Hence the diagnostics of their longitudinal shape has been the subject of an intense research in the last years^22,31–34^.

A well-known and efficient way to measure the shape of a high energy electron bunch consists of probing its Coulomb field, at a short distance *D*. The fundamental time-resolution limit of such a measurement is directly determined by the Coulomb field distribution of a single electron in the laboratory frame (see, e.g., ref. ^35^). The resulting resolution limit is of the order of^9,36^:

17 $${\tau }_{R}^{{\rm{Coulomb}}}\approx \frac{2D}{\gamma c}$$

with *γ* the Lorentz contraction factor, and *c* the speed of light. For energies in the GeV range (i.e., *γ* of the order of several thousands for electrons) and a distance *D* of few millimeters, this time resolution limit ${\tau }_{R}^{{\rm{Coulomb}}}$ lies in the few tens of fs or even below.

As a result, in high energy machines, the main resolution limit is actually set by the capabilities of state-of-art single-shot photonic measurement systems. In particular, Eq. 1 strongly hampered the application range of classical spectrally-decoded electro-optic sampling to values well above the limit set by Eq. 17. The situation is also complicated by the recent trend forward repetition rates in the megahertz-range, in X-ray FELs based on superconducting technology such as FLASH^37^, EuXFEL^4^, and the LCLS-II^38^ and SHINE^39^ projects.

**Fig. 5:**
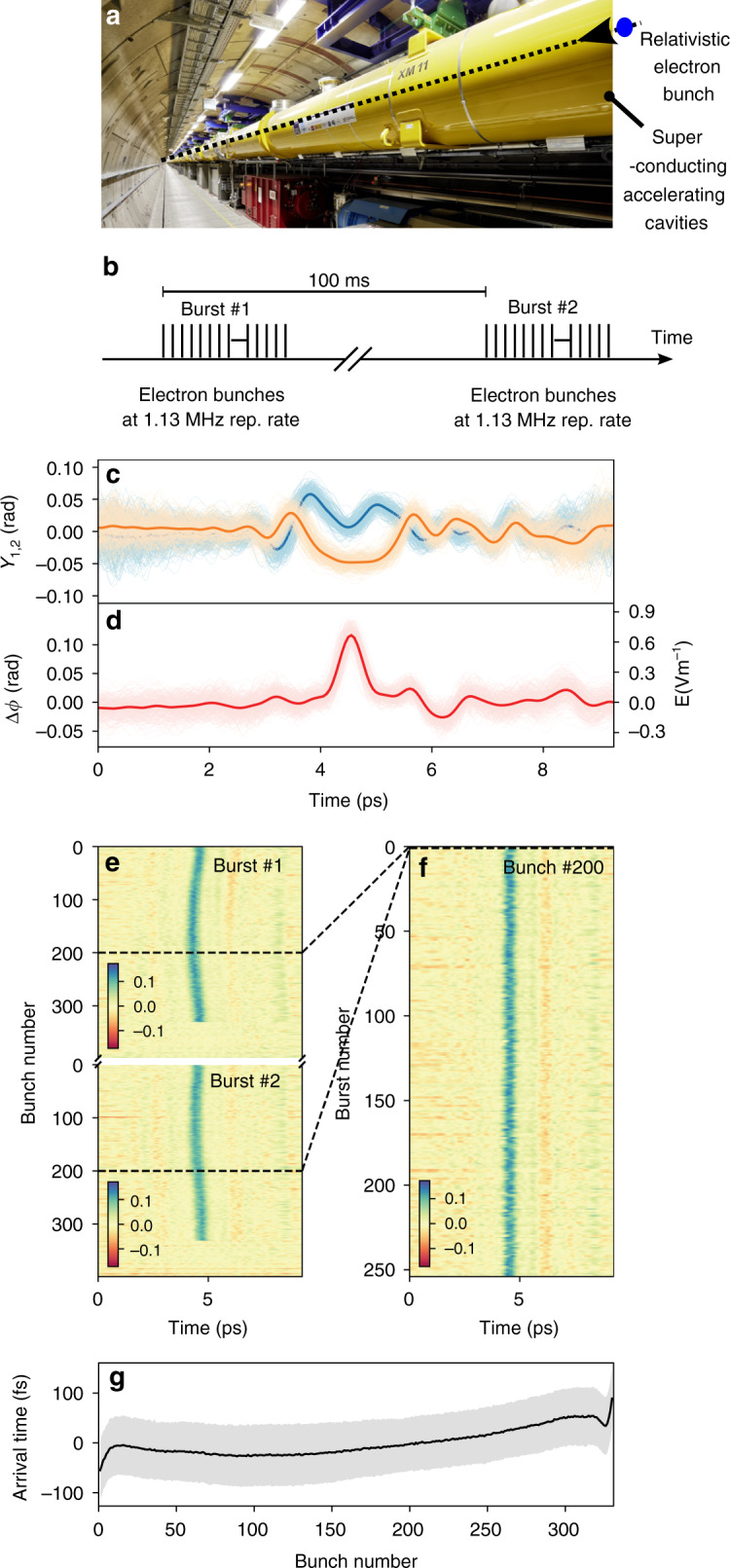
Electron bunch shapes recorded at the European X-ray Free-Electron Laser (EuXFEL). **a** Picture from inside the 3 km-long accelerator tunnel. Our DEOS setup (see Fig. 6) is placed just upstream of the picture, after the first bunch compressor. **b** Timing of the electron bunches in the conditions of the experiment. **c** Electro-optic signals *Y*_1,2_ of a single bunch before reconstruction. **d** Reconstructed electric field. Shaded areas: superposition of single-shot curves, color curves: average over 255 bursts. **e** Electro-optic signal of two bursts (i.e., 800 electron bunches in total). **f** Shape of one bunch (with bunch number 200 within the burst) versus burst number. **g** Arrival time versus bunch number. Shaded areas: RMS arrival time fluctuations, color curve: average over 255 bursts. The EO data are low-pass filtered to 2.5 THz.

In order to test our DEOS reconstruction technique in this context, we realized a proof-of-principle phase-diversity setup at the EuXFEL (Fig. 5a), which is the first hard X-ray FEL operating at megahertz repetition rate^4^ (see also refs. ^40,41^ for examples of applications). In the present case, electron bunches are generated at 1.3 MHz rate in 600 μs long bursts, every 100 ms (Fig. 5b).

**Fig. 6:**
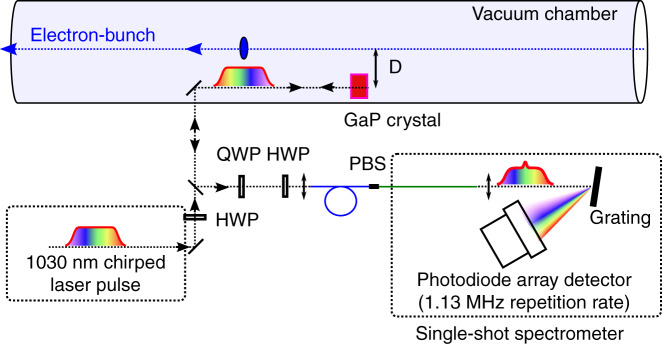
DEOS measurement of the Coulomb field created by relativistic electron bunches at the European X-ray Free-Electron Laser (Eu-XFEL). A laser beam probes the electric field at a distance *D* = 5 mm from the electron beam. HWP: half wave plate, QWP, quarter wave plate, PBS, fiber-based polarizing beam-splitter. Blue lines indicate polarization-maintaining fibers (PM980) and green lines indicate single-mode fibers (HI1060). The probe laser is reflected on a Gallium Phosphide (GaP) crystal back side. The spectrum readout is performed using a KALYPSO linear array camera operating at 1.13 MHz line rate^51,52^. Details of laser transport and KALYPSO focusing optics are not shown—see ref. ^34^ for further details

The setup, based on the single EO channel detection^34^, is depicted schematically in Fig. 6. The EO setup, destined to record the Coulomb field versus time, is located just after the second bunch compressor, where the electron bunches have a beam energy of 700 MeV and a typical RMS duration in the 200 fs range at a charge of 250 pC. The distance *D* between the laser and the electron bunch is of the order of 5 mm which corresponds to a time resolution limit ${\tau }_{R}^{{\rm{Coulomb}}}$ in the 30 fs range for the measurement of the Coulomb field. Note that, in contrast to the previous experiment, where real single-shot operation was achieved, we had here to record each polarization channel *successively*, due to the lack of a second MHz line rate spectrometer (see “Materials and methods”). Hence the present proof-of-principle experiment is not single-shot for the moment. However, a relatively obvious upgrade is planned in order to achieve simultaneous recordings of the two channels.

Results are displayed in Fig. 5c, d. Figure 5c depicts the raw EO signals (after background subtraction and normalization), and Fig. 5d represents the reconstructed electric field. We observe a main peak, for which the duration of 218 fs (RMS) is in very good agreement with the design value of the electron bunch duration at this location and considerably shorter than measured with conventional spectrally decoded EO sampling at the same setup^34^. The smaller negative peak can be attributed to a wakefield following the electron bunch, but the precise interpretation will be the subject of further investigations. Typical examples are displayed in Fig. 5e, f. This measurement system can now be used to perform bunch-by-bunch high resolution measurements of the bunch shape which is crucial information for the control of the bunch compression process.

As previous spectrally decoded EO systems, DEOS also simultaneously measures the arrival time of the electron bunch, which is also a crucial parameter for users of the generated X-rays. The resolution of DEOS for this measurement is expected to be similar to standard EO sampling. In the present case, the arrival time jitter (Fig. 5g) is measured to be 58 fs over a bunch train, which is much lower than the bunch duration (218 fs RMS), and consistent with ref. ^34^.

From the hardware point of view, it is important to note that only few key modifications of our initial EO system^34^ were needed: ensuring a proper (non-standard) orientation of the GaP crystal in the vacuum chamber, and simultaneous detection of the two EO output channels. We thus think that the implementation of a double EO output channel readout will permit to relatively easily adopt the phase diversity-based retrieval method in existing or planned EO diagnostics at FELs or other accelerators.

## Discussion

It is important to note that DEOS is fundamentally different from previous techniques aiming at retrieving the input field using numerical analysis of the EO data (deconvolution^11^, and holography-inspired reconstructions^10^). The presence of zeros (nulls) in the transfer functions *H*_1_(Ω) and *H*_2_(Ω) limits the success of reconstruction methods that do not use the phase diversity technique to relatively narrowband signals, or short analysis windows, if there is no a priori information on properties of the solution. More precisely, deconvolutions or reconstructions from a single channel is ill-posed when the signal spectrum Ω_max_ exceeds the angular frequency Ω_0_ of the first null: ${{{\Omega }}}_{0}=\sqrt{C\pi /2}$ or $\sqrt{3C\pi /2}$ (depending on the channel used) for the Fig. 2 setup. The limit is ${{{\Omega }}}_{0}=\sqrt{C\pi }$ for the case of the more classical situation for which the [-110] axis is parallel to the terahertz field (see Section V of Supplementary Material for the corresponding transfer functions).

In contrast, the DEOS strategy allows a numerical reconstruction to be possible on a frequency range that extends well above the nulls of *H*_1_ and *H*_2_. After applying the MRC algorithm, the resulting transfer function is flat—by design—as shown in Eq. 12. In consequence, DEOS enhances the effective bandwidth over which a THz signal can be reliably recorded without distortion. In practice, the ultimate temporal resolution ${\tau }_{R}^{{\rm{DEOS}}}$ is hence conjectured to be limited by either (1) the laser pulse bandwidth Δ*ν*_*L*_, or (2) the bandwidth Δ*ν*_crystal_ of the EO crystal, whichever is slower. Bandwidth limitations by the electro-optic crystal have been previously extensively studied, and will not be discussed here. The main limitations comes from the phase-matching (i.e., the crystal thickness) and phonon absorption lines, which are at 5.3, 8.0, and 11 THz for classical Zincblende crystals ZnTe, GaAs, and GaP^33^, respectively. Note that the crystal speed limitation affects the response of the Pockels crystal phase shift Δ*ϕ*(*t*) with respect to the input electric field *E*_THz_(*t*).

DEOS specifically enhances the bandwidth and time resolution of the next part of the system, i.e., from the crystal’s output phase shift Δ*ϕ*_in_(*t*) to the final (retrieved) EO signal ${{\Delta }}{\phi }_{{\rm{in}}}^{{\rm{retr}}}(t)$. It is hence natural to introduce a figure of merit that relates the available laser bandwidth (which is a fundamental limitation to the measurement) and the corresponding resolution limitation:

18 $${\tau }_{R}^{{\rm{DEOS}}}={\eta }_{L}\frac{1}{{{\Delta }}{\nu }_{L}}$$

In this Fourier reciprocal relation, the introduced figure of merit *η*_*L*_ has the meaning of a time-bandwidth product. Systematic studies of the resolution versus available laser bandwidth will require specific research, for instance in order to find an analytic bound to *η*_*L*_. However, the resolution ${\tau }_{R}^{{\rm{DEOS}}}$ can already be easily computed numerically in simple cases. As an example, in Table 2 and in Section VIII of the Supplementary Material, we present such a preliminary study in the case of the Eu-XFEL experiment. In this case (and with all widths being defined FWHM) we find that the figure of merit *η*_*L*_ is slightly below unity, and the corresponding time resolution limit (for measuring a Gaussian THz pulse) is approximately 2.2 times the laser pulse duration (see Table 2).

**Table 2:** Example of expected time resolution limitation (numerical simulation) due to the finite laser bandwidth (i.e., an infinitely fast crystal is assumed).

Laser pulse bandwidth	Δ*ν*_*L*_ = 11.5 THz FWHM
Corresponding compressed laser pulse duration	*τ*_*L*_ = 39 fs FWHM
Laser chirped pulse duration at the Pockels crystal	*τ*_*w*_ = 10 ps FWHM
Temporal resolution of the DEOS measurement (computed)	${\tau }_{R}^{{\rm{DEOS}}}=84.5$ fs FWHM
Figure of merit	${\eta }_{L}={{\Delta }}{\nu }_{L}\times {\tau }_{R}^{{\rm{DEOS}}}=0.94$

Typical parameters corresponding to the Eu-XFEL experiment are used, i.e., a laser with 1040 nm wavelength and 40 mn bandwidth FWHM. The THz and laser pulses are taken Gaussian and we consider a chirped pulse duration *τ*_*w*_ = 10 ps. See Section VIII of the Supplementary Material for details, including the dependence of the time resolution ${\tau }_{R}^{{\rm{DEOS}}}$ with respect to the chirped laser probe duration *τ*_*w*_.

In this work, it is interesting to note that, even though DEOS could straighforwardly beat the historical limit set by Eq. 1, we did not have the possibility to produce THz signals presenting features short enough to test the “new” ultimate limit set by the laser bandwidth (Eq. 18), nor the crystal bandwidth limitation. Even in the FEL experiment, the shortest features we could resolve were the bunch shape itself, which has an RMS duration in the 200 fs range, whereas the resolution limit set by the laser bandwidth is numerically predicted (see Table 2) to be less than 100 fs FWHM.

Besides, another equally important property of DEOS lies in the possibility to considerably extend the duration of the analysis window *τ*_*w*_, while keeping the bandwidth of analysis (or time resolution) constant. This is important in particular for time-domain spectrometers (as the one presented in Fig. 3), since the spectral resolution is directly determined by 1/*τ*_*w*_. A long recording window is also crucial in studies of the dynamics of various sources, as THz Free-Electron lasers^3^, Coherent Synchrotron Radiation sources^42^, and quantum cascade lasers^1^. The achievable analysis window *τ*_*w*_ of DEOS is no longer limited by Eq. 1, and this represents a considerable advantage with respect to standard electro-optic sampling. While this suggests that the window of analysis *τ*_*w*_, can be chosen arbitrarily large, component limitations will restrict its achievable range. The most obvious one is the number of pixels *N*_cam_ of the camera that resolves the optical spectrum (1280 and 256 for the first and second experiments of this article). This sets an upper limit to the “effective number of points” that can be recorded to

19 $${N}_{{\rm{eff}}}=\frac{{\tau }_{w}}{{\tau }_{R}} \,<\, {N}_{{\rm{cam}}}$$

Technically, the effective number of points will be also affected by the well-know spectrometer resolution parameters, in particular the number of grating lines that diffracts the laser beam, and aberrations. This will not be discussed here as this does not represent a fundamental limit, and is a well-documented topic. However, when needed, care will have to be taken in the spectrometer choice for reaching an effective number of point *N*_eff_ close to the camera resolution. If applications require an effective number of points *N*_eff_ beyond camera capabilities, different ways may be possible for recording the spectrum. A foreseen alternative is the so-called Dispersive Fourier transform^43^, which may be advantageous from this point of view. This would turn the present recording system to a photonic time-stretch data acquisition system^19^^,^^44^.

Future work aiming at reaching the highest bandwidths and/or longest record duration will probably require specific studies, in particular for managing higher-order dispersion. Indeed, DEOS theory has been established assuming quadratic probe laser chirp, and unwanted third-order dispersion will lead to loss of quality in the reconstruction. It is important to note that the present method implies a fit (see Fig. 4) that also provides an objective measurement of the quality of the model-experiment correspondence. Specific works will be needed when this fit will present strong errors, and this will be expected in extreme cases where strong third-order dispersion is present and the transfer functions *H*_1,2_ present many lobes over the bandwidth of the THz signal. These particular cases will reduce the bandwidth of the measurement (to the range allowing a reasonably good fit). Therefore, we think that an important research direction will consist of extending the DEOS theory in order to take third-order dispersion into account in the reconstruction.

Finally, interesting future directions also concern the quest for the highest possible sensitivity for single-shot DEOS. In this respect, note that the SNR depends on the location inside the laser spectrum, and constant SNR should be achievable by flattening the probe laser spectrum using Fiber Bragg Gratings-based or programmable optical filters (such as the so-called Waveshaper). Furthermore, DEOS is compatible with the recent SNR-enhancement strategies, which has been developed for electro-optic sampling^45^.

In conclusion, we present a novel conceptual framework for spectrally decoded electro-optic sampling, that solves the “temporal resolution problem” open about 20 years ago for terahertz recorders. Technically, the key lies in the derivation of the transfer function, which—in turn—allows well-posed reconstruction algorithms to be possible. In practice, the resulting DEOS design opens the way to terahertz digitizers whose recording length and resolutions is only limited by the probe crystal speed or the laser uncompressed pulse duration. On the short term, one foreseen application of DEOS concerns a revisit of high repetition-rate TDS, up to megahertz rates, and consider monitoring irreversible physical and chemical processes. Another foreseen application is totally opposite, and concerns the applications of very low repetition rate terahertz sources, for which scanning strategies are impractical. This concerns nonlinear TDS using accelerator-based^3,46,47^, as well as table-top-based high power terahertz sources^48^. Future work will include a systematic experimental study of the new limits set by the method in terms of ultimate resolution, time-window, and repetition rate. In this respect, an important direction will consist in combining the present DEOS terahertz recording method with photonic time-stretch^19,42,49^, as this should theoretically allow repetition rate to reach the hundred of megahertz range.

## Materials and methods

### Theoretical details

Proofs are given in the Supplementary Material, and we recall only the main results here. The Pockels-induced phase-shift Δ*ϕ*_in_(*t*) is related to the terahertz electric field by Δ*ϕ*_in_(*t*) = *βE*(*t*) (see Eqs. 7, 8). The factor *β* depends on the relative orientations of the crystal axes and on the polarizations of the laser and terahertz field. For the phase-diversity scheme of Fig. 2 and the setups considered in the main text:

20 $${{\Delta }}{\phi }_{{\rm{in}}}(t)=\beta E(t)$$

21 $$\,{{\mbox{with}}}\,\ \beta =\frac{\pi d}{\lambda }{n}_{0}^{3}{r}_{41}$$

where *n*_0_ is the refractive index at vanishing electric field, *d* the thickness of the crystal and *r*_41_ is its electro-optic coefficient. *λ* is the laser wavelength in vacuum and *E*(*t*) the electric field inside the crystal. See Supplementary Material Section VIII for the values used in the numerical simulations. Note that the value of *β* is two times smaller than for the usual approach used in classical balanced detection (see Eqs. 30 and 49 of the Supplementary Material).

### Experimental recording system for the table-top experiment (Fig. 3)

The laser pulses are delivered by an amplified Sapphire−Titanium laser (Coherent Astrella) with 40 fs duration and 7 mJ output, from which 3 mJ are extracted for this experiment. The emission and detection ZnTe crystals are 110-cut, with 1 mm thickness, and the component orientations are displayed in Table 1. The stretcher is a classical Treacy compressor. The Silicon filter (280 *μ*m thick, at normal incidence) is destined to reject the 800 nm laser light. The imaging spectrometer is composed of a reflection grating (Thorlabs, 1200 lines/mm, blazed at 750 nm), a low-cost 1280 × 1024 pixels monochrome CMOS camera (UI 3240 ML NIR from IDS GmbH) equipped with a 60 mm objective (Nikkor 60 mm F2.8G ED). We also place a cylindrical lens with 100 mm focal length just before the 60 mm lens, in order to spread vertically the optical power onto the CMOS camera. The three spots are vertically binned at analysis stage, thus increasing the equivalent full-well capacity (and the SNR).

At each shot *n*, the recorded image (see Fig. 3b) provides three raw data: the spectra on the two polarization channels *S*_1*n*_(*λ*) and *S*_2*n*_(*λ*) (containing the information on the terahertz field), and the spectrum of the unmodulated laser *S*_0*n*_(*λ*). The camera is trigged by the laser, and acquires 10 images per second. In order to ensure single-shot measurements, we systematically chose a camera exposure time that was shorter than the laser repetition period of 1 ms (the exposure time was 25 *μ*s for the data presented here).

For this test, we did not try to achieve 1 kHz repetition rate (i.e., the repetition rate of the laser), although this type of upgrade would be straightforward using a state-of-art commercial CMOS or CCD camera (as, e.g., in ref. ^50^).

### Data analysis in the table-top experiment

Raw experimental data consist of single-shot camera images (Fig. 3b). At each shot *n*, we extract the three spots: the upper and lower spot corresponding to the EO signals along the two polarizations 1 and 2, and the central spot, which corresponds to the reference laser spectrum without electric field. This latter is used for correcting the shot-to-shot fluctuations of the laser. The three corresponding spectrum are extracted and provide 1-dimensional arrays *S*_1*n*_[*i*], *S*_2*n*_[*i*] and *S*_0*n*_[*i*] (with *i* = 0.. 1279 corresponding to the horizontal camera row index, and physisally to the wavelength *λ*). In addition, we also record beforehand the same data in absence of electric field, which provides the three arrays ${S}_{1}^{{\rm{ref}}}[i]$ and ${S}_{2}^{{\rm{ref}}}[i]$ and ${S}_{0}^{{\rm{ref}}}[i]$.

For obtaining the EO signals *Y*_1*n*_ and *Y*_2*n*_ at each shot *n* (displayed in Fig. 2b) in the following way. We first apply the background subtraction and normalization:

22 $${Y}_{1n}^{{\rm{raw}}}=\frac{{S}_{1n}}{{\sigma }_{1}{S}_{0n}}-1$$

23 $${Y}_{2n}^{{\rm{raw}}}=\frac{{S}_{2n}}{{\sigma }_{2}{S}_{0n}}-1$$

where $${\sigma }_{1,2}=\frac{{S}_{1,2}^{{\rm{ref}}}}{{S}_{0}^{{\rm{ref}}}}$$.

Afterwards, we discard the low-SNR data which correspond to the low intensity wings of the laser spectrum, by multiplying the data by a window function *W*.

24 $${Y}_{1n}[i]=W[i]{Y}_{2n}^{raw}[i]$$

In the experimental results of Fig. 3, we used a simple square window, with *W*[*i*] = 1 for pixels rows *i* = 300 to 1000, and *W*[*i*] = 0 elsewhere.

Note that the first part of the preprocessing (22, 23) involves a measurement of the laser spectrum *S*_0*n*_, so that shot-to-shot laser spectrum fluctuations are canceled out. In other words, *Y*_1,2*n*_ do not depend on the shape of *S*_0*n*_. This can be shown easily, if we assume a fixed relation between the three spectra: *S*_0,1,2*n*_(*λ*) = *f*_0,1,2_(*λ*)*S*_*Ln*_(*λ*), with *S*_*Ln*_(*λ*) the laser spectrum at shot *n*, and assume that *f*_0,1,2_(*λ*) are functions that do not change with time (i.e., they only are determined by the optics transmisions, and adjustment). This preprocessing should theoretically allow the measurements to reach the shot-noise limit.

The obtained *Y*_1,2*n*_ are then used in the MRC retrieval algorithm. Then:

- We apply a Fast Fourier Transform (FFT) to the windowed *Y*_1,2*n*_(*t*) data.
- Apply the MRC formula displayed in Eq. 10.
- Finally apply and inverse FFT for obtaining the reconstructed data ${{\Delta }}{\phi }_{{\rm{in}}}^{{\rm{retr}}}(t)$ and ${E}_{{\rm{in}}}^{{\rm{retr}}}(t)$.

### Other option for the reconstruction

Note that the data processing may be performed in another order. Instead of first applying the background subtraction and normalization (22, 23) before the MRC algorithm, we can also first apply the background subtraction, then MRC, and eventually the normalization. The results are extremely similar for all the experiments presented in this article (see Supplementary Material Section VIII for a reconstruction using this order).

### Experimental setup at EuXFEL (Fig. 6)

The probe pulses are delivered by an amplified Ytterbium fiber laser operating at 1030 nm. The EO effect is achieved in a Gallium Phosphide (GaP) crystal with 2 mm thickness, which is placed inside the vacuum chamber of the accelerator, near the electron beam^34^. The current setup permits to detect only one EO channel output at a time, because one readout camera is available at the time.

The spectra are recorded in single-shot, using a grating spectrometer based on the KALYPSO fast linear array detector^51,52^ operated at a line rate of 1.13 MHz. A first series of electron bunch trains for one polarization is recorded and then the last half-wave plate before the polarizing beam splitter is rotated by *π*/8 to record the complementary polarization. Hence, the two EO channel outputs can be used to reconstruct the individual electron bunch shapes within the burst using the phase-diversity technique. Note that a straightforward upgrade is also planned, with the aim to achieve simultaneous recording of both polarisations, and realize true single-shot operation.

### Data analysis in the EuXFEL experiment

With each burst, we record single-shot spectra on one of the two polarization directions *S*_1*n*_(*λ*), *S*_2*n*_(*λ*) and additionally unmodulated spectra with laser and no electron bunch ${S}_{1,2}^{{\rm{no}}\ {\rm{bunch}}}$, and the spectra without laser ${S}_{1,2}^{{\rm{dark}}}$.

However as we do not record the unmodulated laser spectra *S*_0*n*_(*λ*) of the same laser pulses in the XFEL experiment, we cannot compensate for the shot-to-shot fluctuations of the laser faster than about 2 kHz. The data analysis is then similar to the 800 nm experiment case. We thus define the EO signals (before applying the MRC reconstruction) as:

25 $${Y}_{1n}^{{\rm{raw}}}=\frac{{S}_{1n}-{S}_{1}^{{\rm{dark}}}}{{S}_{1}^{{\rm{no}}\ {\rm,{bunch}}}-{S}_{1}^{{\rm{dark}}}}-1$$

26 $${Y}_{2n}^{{\rm{raw}}}=\frac{{S}_{2n}-{S}_{2}^{{\rm{dark}}}}{{S}_{2}^{{\rm{no}}\ {\rm{bunch}}}-{S}_{2}^{{\rm{dark}}}}-1$$

And as before, we multiply the data by a window function (see Eq. 24):

27 $${Y}_{1,2n}[i]=W[i]{Y}_{1,2n}^{{\rm{raw}}}[i]$$

We chose here a Tukey function for *W*[*i*], which falls to zero at the points where the laser spectrum is at 5% of its maximum, and with a shape parameter equal to 0.25. Then we apply the MRC algorithm in exactly the same way than for the previously described table-top experiment.

## Supplementary information

Supplemantal Material
