# Supplementary material for: Phase Diversity Electro-optic Sampling: A new approach to single-shot terahertz waveform recording
Source: Light Sci Appl. 2022 Jan 10;11:14. doi: 10.1038/s41377-021-00696-2 (PMC8748811; doi:10.1038/s41377-021-00696-2)
Supplement: Supplementary file 1 — Supplemantal Material [file 41377_2021_696_MOESM1_ESM.pdf]

Phase Diversity Electro-optic Sampling: A new approach to single-shot terahertz waveform recording

Eléonore Roussel[1], Christophe Szwaj[1], Bernd Steffen[2], Clément Evain[1], Christophe Gerth[2], Bahram Jalali[3], and Serge Bielawski[1]

[1] *Univ. Lille, CNRS, UMR 8523 - PhLAM - Physique des Lasers, Atomes et Molécules, Centre d'Étude Recherches et Applications (CERLA), F-59000 Lille, France.*

[2] *DESY (Deutsches Elektronen-Synchrotron), Notkestr. 85, D-22607 Hamburg, Germany*

[3] *Electrical and Computer Engineering Department, University of California, Los Angeles, 420 Westwood Plaza, Los Angeles, CA 90095, USA*

(Dated: November 23, 2021)

SUPPLEMENTARY MATERIAL: DERIVATION OF THE TRANSFER FUNCTIONS H_1 AND H_2

The derivation of the transfer functions $H_1(\Omega)$ and $H_2(\Omega)$ is decomposed in the following steps:

- In Section I, we recall the principle of the spectrally-encoded detection and derive the relationship between the time and spectral domain.
- In Section II, we recall the dependence between the crystal orientation and the resulting properties of the field-induced birefringence.
- In Section III, we summarize the physical arguments that underly the choices made in experimental setups.
- In Section IV, we present the Jones matrix modeling of the measurement system.
- In Section V, we derive the transfer functions in the case of the classic arrangement found in previous publications (and show why a deconvolution is impossible in this case).
- In Section VI, we derive the transfer functions H_1 and H_2 when using the special arrangement of the article, which allows the input to be retrieved using phase diversity, and the MRC algorithm.
- In Section VIII, we present the numerical calculations that provide an estimate to the time-resolution limit of DEOS.

In this work, we consider an infinitely fast electro-optic crystal, and we assume that the crystal does not display any imperfections, as residual birefringence (due, e.g., to stress).

I. SPECTRALLY-ENCODED DETECTION: RELATION BETWEEN THE INPUT TIME AND THE OPTICAL FREQUENCY AT THE SPECTROMETER

In the spectrally-encoded electro-optic detection, an input signal is encoded in a linearly chirped laser pulse that has the form:

$$E_0(t) = A(t)e^{-i\frac{C}{2}t^2}, \tag{1}$$

where $A(t)$ is a slowly varying envelope where we have omitted the carrier frequency and $C = \partial\omega/\partial t$ is the chirp rate of the laser.

The laser pulse is modulated (temporally) by the electric field under interest. Then, since the laser pulse is chirped, one expects to observe the electric field-induced modulation in the optical spectrum. We derive here the relation between the input time t and the optical spectrometer's frequency ω.

The spectrum of the laser is given by the Fourier transform:

$$\tilde{E}_0(\omega) = \int_{-\infty}^{+\infty} A(t)e^{-i\frac{C}{2}t^2}e^{-i\omega t}dt, \tag{2}$$

and can be written as a convolution:

$$\tilde{E}_0(\omega) = \frac{1}{2\pi}\int_{-\infty}^{+\infty}\tilde{A}(\omega')\times\left[\sqrt{-\frac{i2\pi}{C}}e^{i\frac{(\omega-\omega')^2}{2C}}\right]d\omega', \tag{3}$$

where $\tilde{A}(\omega)$ is the Fourier transform of $A(t)$. Thus,

$$\tilde{E}_0(\omega) = \sqrt{-\frac{i2\pi}{C}}\frac{1}{2\pi}\int_{-\infty}^{+\infty}\tilde{A}(\omega')\times e^{i\frac{\omega^2-2\omega\omega'+\omega'^2}{2C}}d\omega', \tag{4}$$

$$= \sqrt{-\frac{i2\pi}{C}}e^{i\frac{\omega^2}{2C}}\times\frac{1}{2\pi}\int_{-\infty}^{+\infty}\left[e^{i\frac{\omega'^2}{2C}}\tilde{A}(\omega')\right]e^{i\left(-\frac{\omega}{C}\right)\omega'}d\omega'. \tag{5}$$

As $A(t)$ is slowly varying, $\tilde{A}(\omega')$ is peaked around $\omega' \approx 0$ and the exponential term can be replaced by:

$$e^{i\frac{\omega'^2}{2C}} \approx 1. \tag{6}$$

One can expand Eq. (5) around $\omega' = 0$, and obtain:

$$\tilde{E}_0(\omega) \approx \sqrt{-\frac{i2\pi}{C}}e^{i\frac{\omega^2}{2C}}\times A\left(-\frac{\omega}{C}\right). \tag{7}$$

The time evolution of the laser pulse $|A(t)|^2$ is transposed into the spectrum

$$|\tilde{E}_0(\omega)|^2 = \frac{2\pi}{C}\left|A\left(-\frac{\omega}{C}\right)\right|^2, \tag{8}$$

with the input time coordinate t related to the output spectral coordinate ω by:

$$t \equiv -\frac{\omega}{C}. \tag{9}$$

II. KNOWN RELATION BETWEEN BIREFRINGENCE AND ELECTRIC FIELD ORIENTATION

We remind here the relationship between the electric field orientation, and the birefringence which is induced in the Pockels crystal. This properties will directly determine the transfer functions as we will show in the next Sections (and will thus determine the necessary condition for input retrieval).

The theory described below applies to Pockels crystals of the Zinc blende type, i.e., typically ZnTe, GaP or GaAs. We consider a (110)-cut crystal as shown in Fig. S1a. The THz electric field is the (110)-plane with its linear polarization at an angle α with respect to the [-110] direction.

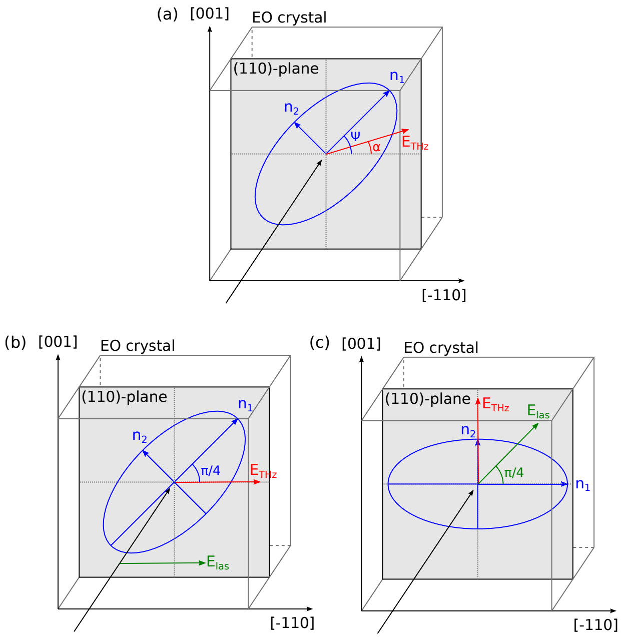

FIG. S1. (a) The (110)-plane of a zinc blende crystal. The THz electric field pulse impinges perpendicular to this plane and its electric vector (red) forms an angle α with the [-110] axis. The angle ψ between the long axis of the induced refractive index ellipsoid (blue) and the [-110]-axis is α-dependent. (b) Geometry used in the standard EO configuration: the laser and the THz polarization are parallel to the [-110]-axis. (b) Geometry used in the phase diversity configuration: the THz polarization is perpendicular to the [-110]-axis and the laser polarization is set at 45 degrees with respect to the [-110]-axis.

The properties of the field-induced birefringence are known [1]. The birefringence is uniaxial. The slow and fast axes lie in the (110)-plane, and the n_1 direction makes an angle ψ (see Figure S1) with respect to the [-110] axis:

$$\cos(2\psi) = \frac{\sin\alpha}{\sqrt{1+3\cos^2\alpha}}. \tag{10}$$

The refractive indices n_1 and n_2 are given by:

$$n_1 = n_0 + \eta_1(\alpha)E_{THz}, \tag{11}$$
$$n_2 = n_0 + \eta_2(\alpha)E_{THz}, \tag{12}$$

with

$$\eta_1(\alpha) = \frac{n_0^3 r_{41}}{4}\left(\sin\alpha + \sqrt{1+3\cos^2\alpha}\right), \tag{13}$$
$$\eta_2(\alpha) = \frac{n_0^3 r_{41}}{4}\left(\sin\alpha - \sqrt{1+3\cos^2\alpha}\right), \tag{14}$$

where n_0 is the refractive index in the absence of electric field, r_{41} is the electro-optic coefficient of the crystal and E_{THz} is the instantaneous value of the THz electric field.

In the frame of the birefringence axes, the Jones matrix of the crystal read:

$$\mathbf{M}_{Xtal} = \begin{pmatrix} e^{i\phi_1(\alpha,E_{THz})} & 0 \\ 0 & e^{i\phi_2(\alpha,E_{THz})} \end{pmatrix}, \tag{15}$$

where

$$\phi_1(\alpha, E_{THz}) = \frac{2\pi d}{\lambda_0}\eta_1(\alpha)E_{THz}, \tag{16}$$
$$\phi_2(\alpha, E_{THz}) = \frac{2\pi d}{\lambda_0}\eta_2(\alpha)E_{THz}, \tag{17}$$

and λ_0 is the laser wavelength in vacuum, and d is the crystal thickness. Note that we have omitted the constant factor $e^{i\phi_0} = e^{2\pi n_0 d/\lambda_0}$, as it will not play a role in the following.

As an important point, the Pockels effect creates phase modulations ϕ_1 and ϕ_2 which have different values along the two birefringence directions. Furthermore it is possible to adjust the ratio between ϕ_1 and ϕ_2 by choosing the angle α between the THz electric field and the crystal axes. This will be a crucial point for the phase diversity-enabled reconstruction.

III. STRATEGY

We consider the experimental layout displayed in Figure S2. We will not perform a systematic search for situations leading to adequate transfer functions, given the number of parameters. Instead, we will limit ourselves to two particularly relevant situations:

- We first consider the situation classically used in most balanced detection electro-optic sampling setups, which is optimized for obtaining a maximal phase difference $\phi_1 - \phi_2$. This consists in applying

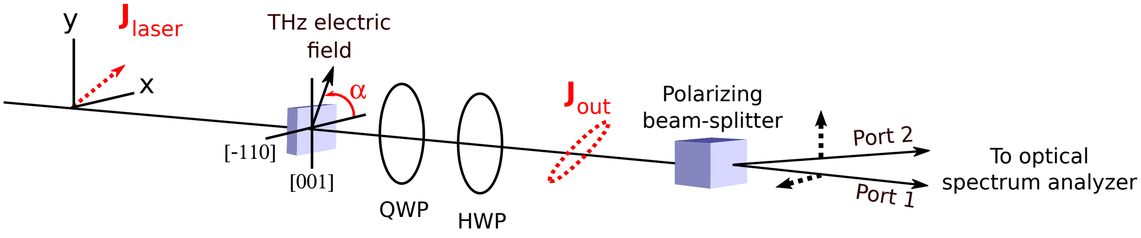

FIG. S2. Setup arrangement corresponding to the Jones matrix calculations. The Jones matrix of the system is calculated from $\mathbf{J}_{laser}$ to $\mathbf{J}_{out}$. Without loss of generality, we assume that the [-110] axis of the crystal is in the horizontal direction. Many orientations choices are possible for the waveplates, crystal as well as the laser and terahertz polarizations. However once the angle α is fixed, all other orientations (of J_{laser}, QWP and HWP) are determined by the constraints described in Section IV A. Note that the birefringence axes of the crystal do NOT generatlly coincide with the crystallographic axes [-110] and [001] (see Section II).

the THz electric field along the [-110] direction (see, e.g., [2]):

$$\alpha = 0, \tag{18}$$

$$\phi_2 = -\phi_1 \tag{19}$$

- Then we consider the situation of the article, which is inspired by the work on time-stretch using Mach-Zehnder modulators [3]. This consists in applying the electric field along the [001] direction:

$$\alpha = \pi/2 \tag{20}$$

$$\phi_1 \neq 0 \tag{21}$$

$$\phi_2 = 0. \tag{22}$$

We will derive the transfer functions in the two cases. We will also show that only the second one will be compatible with the phase diversity-enabled reconstruction described in the article.

Finally, instead of using directly the electric field E_{THz}, it will be convenient to use a dimensionless variable. We choose here the phase difference $\Delta\phi$ as the input variable for the transfer functions:

$$\Delta\phi = \phi_1 - \phi_2 = \frac{2\pi d}{\lambda_0}\left[\eta_1(\alpha) - \eta_2(\alpha)\right] E_{THz}, \tag{23}$$

or:

$$\Delta\phi = \phi_1 - \phi_2 = \frac{2\pi}{\lambda_0}\frac{n_0^3}{2}\sqrt{1 + 3\cos\alpha} E_{THz}. \tag{24}$$

Note that the coefficient linking $\Delta\phi$ and the electric field E_{THz} depends on α.

IV. JONES MATRIX MODELING OF THE SYSTEM

A. Experimental layout and constraints on the angles

The general setup is represented in Figure S2. Except for the special choice of the angle α, we use the same adjustement strategy as for "classical" balanced detection electro-optic sampling:

- For the calculation (and without loss of generatltity) we choose the [-110] axis horizontal in all situations.
- The laser polarization is chosen linear and at 45 degrees with respect to the birefringence axes of the Pockels crystal (note that the birefringence axes do not generally coincide with the crystallographic axes – see Eq. 10).
- We measure the ellipticity after the crystal, using a quarter-wave plate associated with a polarizer. The quarter-wave plate axes hence coincide with the crystal's axes, and the polarizer detects the power components at 45 degrees with respect to the crystal's axes. Note that the role of the optional half-wave plate is just to allow this measurement to be made, while keeping the polarizer's axes in the horizontal and vertical directions.

This choice imposes strong constraints on most angles. Once the angle α between the THz field direction and the crystal axes has been fixed, all other angle are determited (modulo 90 degree rotations for the QWP and 45 degree rotations for the HWP).

In other words, using this strategy, α becomes the only free parameter (if we exclude trivial symmetries in the crystal, waveplates, and laser polarization).

B. General expression of Jones matrices

The first step consists of writing the the Jones matrix from the crystal input to the entrance of the polarizer.

$$\mathbf{J}_{out} = \mathbf{M}\mathbf{J}_{laser}, \tag{25}$$

with

$$\mathbf{M} = \mathbf{M}_{HWP}(\theta_{HWP})\mathbf{R}^{-1}(\psi)\mathbf{M}_{QWP}\mathbf{M}_{Xtal}\mathbf{R}(\psi), \tag{26}$$

and:

$$\mathbf{M_{QWP}} = \begin{pmatrix} e^{-i\frac{\pi}{4}} & 0 \\ 0 & e^{i\frac{\pi}{4}} \end{pmatrix}, \tag{27}$$

$$\mathbf{M_{HWP}}(\theta_{\mathbf{HWP}}) = \mathbf{R^{-1}}(\theta_{\mathbf{HWP}}) \begin{pmatrix} e^{-i\frac{\pi}{2}} & 0 \\ 0 & e^{i\frac{\pi}{2}} \end{pmatrix} \mathbf{R}(\theta_{\mathbf{HWP}}) \tag{28}$$

$$\theta_{HWP} = (\psi + \pi/4)/2 \text{ or } (\psi - \pi/4)/2, \tag{29}$$

where $\mathbf{M}_{Xtal}$ is given by Eq. (15). $\mathbf{R}(\theta)$ stands for the rotation matrix by an angle θ. θ_{HWP} correspond to the orientation angle of the half wave plate. Note that the birefringence axes of the Pockels crystal are tilted by an angle ψ with respect to the [-110] and [001] crystallographic axes (see Section II).

V. TRANSFER FUNCTIONS IN THE STANDARD ELECTRO-OPTIC SAMPLING ARRANGEMENT $\alpha = 0$

In this Section, we derive the transfer functions $H_1(\Omega)$ and $H_2(\Omega)$ in the case of the traditional arrangement displayed in Figure S3a. The adjustments are such that:

- $\alpha = 0$, i.e., the [-110] crystallographic is oriented parallel to the THz field.
- We assume that the [-110] axis is horizontal for the calculation.

Given the strategy chosen in Section IV A, this implies that:

- The birefringence axes of the crystal are at 45 degrees with respect to the horizontal direction [since $\psi = 45$ degree, see Eq. (10)].
- The quarter wave plate axes have the same orientation than the crystal birefringence axes, i.e., at 45 degrees with respect horizontal
- The laser polarization should be horizontal or vertical. We choose a horizontal direction here.
- The half-wave plate is not needed.

The input variable of our problem is defined as the phase shift $\Delta\phi$ induced by the electric field E_{THz}. In the present case for which $\alpha = 0$ (see Eq. 24), it is related to the electric field E_{THz} by:

$$\Delta\phi = \Delta\phi^{std} = \frac{2\pi d}{\lambda_0} \cdot n_0^3 r_{41} E_{THz}(t). \tag{30}$$

A. Jones matrix calculation

The Jones matrix of the whole system (down to the polarizer's entrance) can be written:

$$\mathbf{M} = \mathbf{M}^{std} = \mathbf{R}^{-1}(\frac{\pi}{4})\mathbf{M}_{QWP}\mathbf{M}_{Xtal}\mathbf{R}(\frac{\pi}{4}), \tag{31}$$

where we have skipped the half-wave plate, and $\mathbf{M}_{Xtal}$ is given by Eq. (15).

The Jones vector of the input laser is:

$$\mathbf{J}^{std}_{laser} = E_0(t)\begin{pmatrix} 1 \\ 0 \end{pmatrix}, \tag{32}$$

where $E_0(t)$ is the complex electric field of the laser.

The output Jones vector is:

$$\mathbf{J}^{std}_{out} = \mathbf{M}^{std}\mathbf{J}^{std}_{laser} = \begin{pmatrix} E_1^{std}(t) \\ E_2^{std}(t) \end{pmatrix} \tag{33}$$

$$= \frac{E_0(t)}{\sqrt{2}} \cdot \begin{pmatrix} \cos\left(\frac{\Delta\phi^{std}(t)}{2}\right) + \sin\left(\frac{\Delta\phi^{std}(t)}{2}\right) \\ i\left[\cos\left(\frac{\Delta\phi^{std}(t)}{2}\right) - \sin\left(\frac{\Delta\phi^{std}(t)}{2}\right)\right] \end{pmatrix} \tag{34}$$

$$= \frac{E_0(t)}{2\sqrt{2}} \cdot \begin{pmatrix} (1+i)\left(-ie^{i\frac{\Delta\phi^{std}(t)}{2}} + e^{-i\frac{\Delta\phi^{std}(t)}{2}}\right) \\ i(1+i)\left(e^{i\frac{\Delta\phi^{std}(t)}{2}} - ie^{-i\frac{\Delta\phi^{std}(t)}{2}}\right) \end{pmatrix}. \tag{35}$$

The two components of this Jones vector correspond to the two complex fields at the optical spectrum analyzer's inputs (i.e. at port 1 and port 2).

B. Note: Analogy with previous work on Mach-Zehnder modulators

A this stage, it is interesting to note that the complex electric fields at the two outputs [Eqns. (35)] take the same form as for the Mach-Zehnder modulator displayed in Figure S3b (see Ref. [3]). In this work, which has been

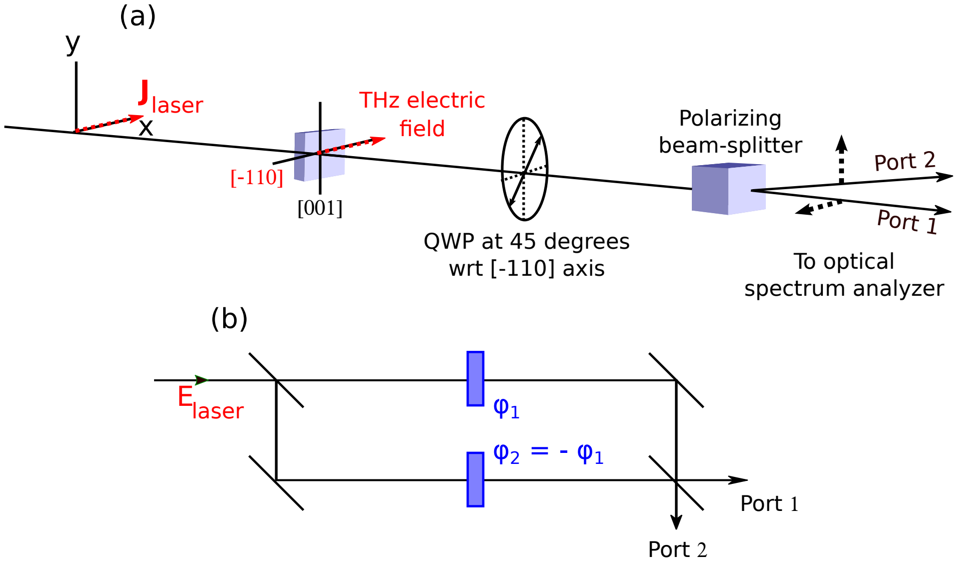

FIG. S3. (a) Optical component orientations for the standard EO configuration (i.e., NOT considered in the article). The laser and THz polarizations are parallel to the [-110] axis. Note that the biregringence axes of the crystal are at 45 degrees with respect to the [-110] crystallographic axis (see Fig. S1b). The polarizer input directions are along the [-110] and [001] axes. (b) Analogy with a symmetric Mach-Zehnder modulator, i.e., for which the phase modulations are equal and in opposite phase.

performed in the context of photonic time-stretch of RF signals, the authors found transfer functions of the form:

$$H_{1,2}\left(\nu\right) = \pm\cos\left(D\nu^2\right), \tag{36}$$

with ν the frequency of the input signal and D a parameter that depends on the laser chirp and setup details (see Ref. [3] for details).

Although the readout method is different in our case (an optical spectrum analyzer), we may reasonably conjecture a similar transfer function. We will show that it is indeed the case in the following.

C. Calculation of the transfer functions for the standard electro-optic sampling configuration

1. Small signal approxiation

For a small phase modulation (i.e. $\Delta\phi^{std}(t) \ll 1$), the two polarization components can be approximated by

$$\begin{pmatrix} E_1^{std}(t) \\ E_2^{std}(t) \end{pmatrix} \approx \frac{E_0(t)}{\sqrt{2}} \cdot \begin{pmatrix} 1 + \frac{\Delta\phi^{std}(t)}{2} \\ i\left[1 - \frac{\Delta\phi^{std}(t)}{2}\right] \end{pmatrix}. \tag{37}$$

According to the Appendix (Section VII) it is useful to rewrite Eq. (37) in the form:

$$\begin{pmatrix} E_1^{std}(t) \\ E_2^{std}(t) \end{pmatrix} = E_0(t) \cdot \begin{pmatrix} T_1^{std}(t) \\ T_2^{std}(t) \end{pmatrix}, \tag{38}$$

with the two transmission functions:

$$T_1^{std}(t) = \mathcal{T}_1\left[1 + m_1 e^{i\psi_1}\Delta\phi^{std}(t)\right], \tag{39}$$
$$T_2^{std}(t) = \mathcal{T}_2\left[1 + m_2 e^{i\psi_2}\Delta\phi^{std}(t)\right], \tag{40}$$

with $\mathcal{T}_1 = 1/\sqrt{2}$, $\mathcal{T}_2 = i/\sqrt{2}$, $m_1 = 1/2$, $m_2 = -1/2$ and $\psi_{1,2} = 0$.

2. Calculation in Fourier space: derivation of the transfer functions

The experimental data consist of the optical spectra (recorded using an optical spectrum analyzer), after background subtraction and normalization by the laser spectrum. Hence we define:

$$Y_1(t) \equiv \frac{|\tilde{E}_1^{std}(\omega)|^2 - |\mathcal{T}_1\tilde{E}_0(\omega)|^2}{|\mathcal{T}_1\tilde{E}_0(\omega)|^2}, \tag{41}$$

$$Y_2(t) \equiv \frac{|\tilde{E}_2^{std}(\omega)|^2 - |\mathcal{T}_2\tilde{E}_0(\omega)|^2}{|\mathcal{T}_2\tilde{E}_0(\omega)|^2}, \tag{42}$$

where the input time t appearing in the $Y_1(t)$ and $Y_2(t)$ corresponds to the OSA's optical frequency ω according to Eq. (9) (see Section I).

The final step consists in finding the relations between the input signal $\Delta\phi^{std}(t)$ and the outputs $Y_{1,2}(t)$. This relation is relatively easily found in Fourier space (see Section VII). We find that

$$\tilde{Y_1}(\Omega) = H_1^{std}(\Omega)\Delta\tilde{\phi}^{std}(\Omega) \tag{43}$$
$$\tilde{Y_2}(\Omega) = H_2^{std}(\Omega)\Delta\tilde{\phi}^{std}(\Omega) \tag{44}$$

where $\tilde{Y}_{1,2}$ and $\Delta\tilde{\phi}^{std}$ are the Fourier transforms of $Y_{1,2}$ and $\Delta\phi^{std}$ respectively. Using Eqs. (60, 71), we find:

$$H_1^{std}(\Omega) = \cos\left(\frac{\Omega^2}{2C}\right) \tag{45}$$

$$H_2^{std}(\Omega) = -\cos\left(\frac{\Omega^2}{2C}\right) \tag{46}$$

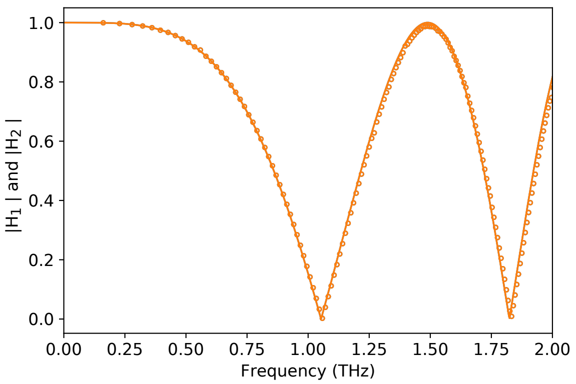

FIG. S4. Norms of the transfer functions $|H_1^{std}(\Omega)|$ and $|H_2^{std}(\Omega)|$ for the standard electro-optic configuration $\alpha = 0$. Dots: numerical results, lines: analytical results. Note that $|H_1^{std}(\Omega)|$ and $|H_2^{std}(\Omega)|$ are identical.

The transfer functions are shown in Figure S4. We can show clearly the existence of zeros at particular frequencies, which explains why a deconvolution is not possible when the terahertz signal spectrum spreads above a critical value. We can also see that H_1^{std} and H_2^{std} are identical. Hence the phase diversity technique and Maximum Ratio Combining (MRC) algorithms can not be applied in this case.

VI. TRANSFER FUNCTIONS FOR THE ARRANGEMENT USED IN THE ARTICLE: $\alpha = \pi/2$ CASE

A. Intuition from previous time-stretch work

We need now to find a situation for which the transfer functions are different on the two outputs of the polarizer. A natural attempts consists in making the same calculation as for the previous Section, in the $\alpha \neq 0$ case.

However we may anticipate that this should work, by considering in previous works on time-stretch using a Mach-Zehnder modulator. Different transfer functions were obtained in the MZ modulator by using an electro-optic crystal in one arm only [3], as shown in Fig. S5b. In that configuration, the transfer functions on the two outputs are of the form:

$$H_{1,2}(\nu) = \pm\cos\left(D\nu^2 \pm \frac{\pi}{4}\right), \tag{47}$$

where the zeros of the functions are interleaved, thus leading to a well-posed deconvolution problem. We will see that this situation will be obtained in our system for $\alpha = \pi/2$.

B. Component orientations

The adjustments are such that (see Figure S5a):

- $\alpha = \pi/2$, i.e., the THz field is now perpendicular to the [-110] crystallographic axis.
- We assume that the [-110] axis is horizontal in the calculation.

We require the same contraints than for Section IV A, as this is expected to provide the highest electro-optic modulation. This leads to the following situation:

- The birefringence axes of the crystal are horizontal and vertical [since $\psi = 0$, see Eq. (10)].
- The laser polarization is taken at a 45 degree angle with respect to horizontal (i.e., at 45 degrees from the crystal's optical axes).
- The quarter wave plate axes have the same orientation as those of the crystal (horizontal and vertical).
- The analysis directions should be made at 45 degrees with respect to the input laser polarization. We thus use the half-wave at 22.5 degrees with respect to horizontal.

As a main difference with the previous Section (i.e., the $\alpha = 0$ case), there is an asymmetry between the two index modulations in the electro-optic crystal:

$$\mathbf{M}_{Xtal}^{phd} = \mathbf{M}_{\phi}^{phd} = \begin{pmatrix} e^{i\phi_1} & 0 \\ 0 & 1 \end{pmatrix}, \tag{48}$$

It is also important to note that our input variable $\Delta\phi$, which will be called $\Delta\phi^{phd}$ is now related the THz electric field by (see Eq. 24):

$$\Delta\phi^{phd}(t) = \phi_1(t) = \frac{\pi d}{\lambda_0} \cdot n_0^3 r_{41} E_{THz}(t). \tag{49}$$

Note that the THz-induced birefringence is now *half the birefringence in the standard $\alpha = 0$ situation* [Eq. (30)].

C. Jones matrix calculations

The Jones matrix of the whole system (down to the polarizer's entrance) is now:

$$\mathbf{M} = \mathbf{M}^{phd} = \mathbf{M}_{HWP}(\pi/8)\mathbf{M}_{QWP}\mathbf{M}_{Xtal}, \tag{50}$$

The Jones vector of the input laser is now:

$$\mathbf{J}_{laser}^{phd} = E_0(t)\begin{pmatrix} 1/\sqrt{2} \\ 1/\sqrt{2} \end{pmatrix}, \tag{51}$$

where $E_0(t)$ is the complex electric field of the laser.

The output Jones vector is:

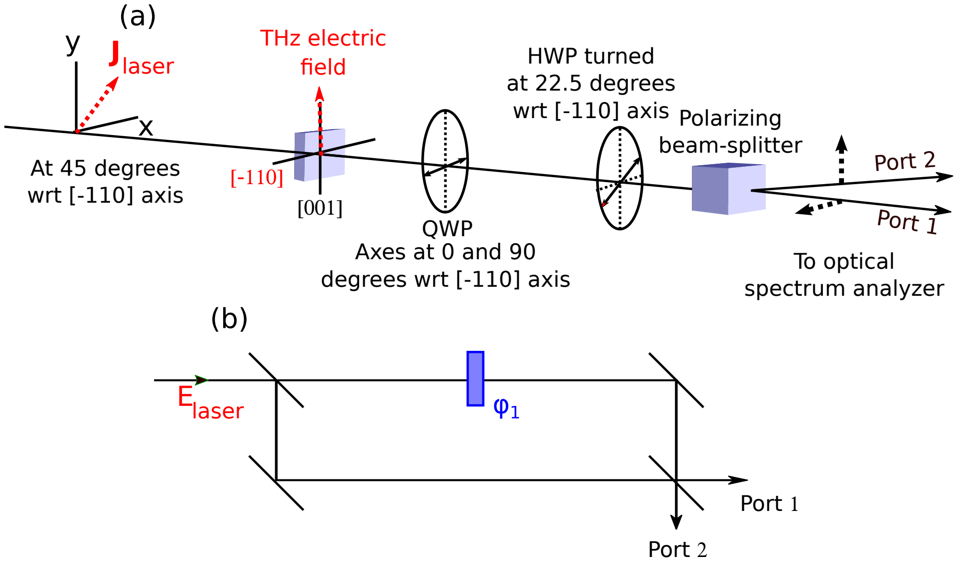

FIG. S5. (a) Axes orientations for the phase-diversity-based EO configuration considered in the article. As a key point, the THz and laser polarizations are perpendicular and at 45 degrees to the [-110] axis, respectively. Note that the birefringence axes of the crystal are along the [-110] and [001] crystallographic axes (see Fig. S1c) in contrast to the Fig. S3 case. The polarizer input directions are along the [-110] and [001] axes. (b) Analogy with a Mach-Zehnder modulator, for which the phase modulation is applied one arm only.

$$\mathbf{J}_{out}^{phd} = \mathbf{M}^{phd}\mathbf{J}_{laser}^{phd} = \begin{pmatrix} E_1^{phd}(t) \\ E_2^{phd}(t) \end{pmatrix} = \frac{E_0(t)}{2\sqrt{2}} \cdot \begin{pmatrix} -(1+i)\left(e^{i\Delta\phi^{phd}(t)} - i\right) \\ (1+i)\left(e^{i\Delta\phi^{phd}(t)} + i\right) \end{pmatrix} \tag{52}$$

The two components of this Jones vector correspond to the two complex fields at the optical spectrum analyzer's inputs.

D. Transfer functions in the phase diversity configuration: results

We perform the same calculations as in Section V, using the present configuration.

For small phase modulation (i.e. $\Delta\phi^{phd}(t) \ll 1$), the two polarization components can be approximated by

$$\begin{pmatrix} E_1^{phd}(t) \\ E_2^{phd}(t) \end{pmatrix} \approx \frac{E_0(t)}{\sqrt{2}} \cdot \begin{pmatrix} -1 + (1-i)\frac{\Delta\phi^{phd}(t)}{2} \\ i - (1-i)\frac{\Delta\phi^{phd}(t)}{2} \end{pmatrix}. \tag{53}$$

According to Appendix VII, it is useful to rewrite Eq. (53) in the form:

$$\begin{pmatrix} E_1^{phd}(t) \\ E_2^{phd}(t) \end{pmatrix} = E_0(t) \cdot \begin{pmatrix} T_1^{phd}(t) \\ T_2^{phd}(t) \end{pmatrix}, \tag{54}$$

with the two transmission functions:

$$T_1^{phd}(t) = \mathcal{T}_1\left[1 + m_1 e^{i\psi_1}\Delta\phi^{phd}(t)\right], \tag{55}$$

$$T_2^{phd}(t) = \mathcal{T}_2\left[1 + m_2 e^{i\psi_2}\Delta\phi^{phd}(t)\right], \tag{56}$$

with $\mathcal{T}_1 = -1/\sqrt{2}$, $\mathcal{T}_2 = i/\sqrt{2}$, $m_1 = \sqrt{2}/2$, $m_2 = \sqrt{2}/2$, $\psi_1 = 3\pi/4$ and $\psi_2 = \pi/4$.

Hence, using Eqs. (60, 71), the transfer functions of a setup with such transmission functions are:

$$H_1^{phd}(\Omega) = -\sqrt{2}\cos\left(\frac{\Omega^2}{2C} - \frac{\pi}{4}\right) \tag{57}$$

$$H_2^{phd}(\Omega) = \sqrt{2}\cos\left(\frac{\Omega^2}{2C} + \frac{\pi}{4}\right) \tag{58}$$

The functions $H_1^{phd}(\Omega)$ and $H_2^{phd}(\Omega)$ correspond to the functions $H_1(\Omega)$ and $H_2(\Omega)$ of the article.

The transfer functions are shown in Fig. S6. It is clearly visible that the two output ports present interleaved zeros that permit to perform a deconvolution to retrieve the input signal.

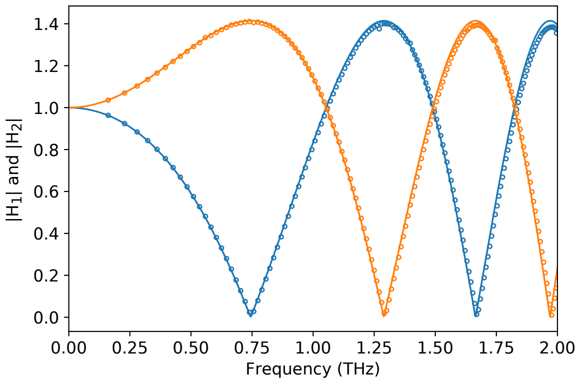

FIG. S6. Norms of the transfer functions $|H_1^{phd}(\Omega)|$ and $|H_2^{phd}(\Omega)|$ for the phase-diversity electro-optic configuration $\alpha = \pi/2$. Dots: numerical results, lines: analytical results.

VII. APPENDIX: TECHNICAL DETAILS OF THE TRANSFER FUNCTION CALCULATION

We consider that the input signal $X_{in}(t)$ modulates the chirped laser pulse in phase and amplitude:

$$E(t) = T(t)E_0(t) \tag{59}$$

with $T(t) = \mathcal{T}\left[1 + m_a X_{in}(t)\right] e^{i m_\phi X_{in}(t)}$ the transmission function, $\mathcal{T}$ is generally complex and $m_{a,\phi}$ the amplitude and phase modulations.

For small modulation, the transmission function can be simplified in the form:

$$T(t) = \mathcal{T}\left[1 + m e^{i\psi} X_{in}(t)\right], \tag{60}$$

with $m_a = m\cos\psi$ and $m_\phi = m\sin\psi$.

The input signal is chosen to be monochromatic,

$$X_{in}(t) = a\cos\left(\Omega t + \phi\right). \tag{61}$$

The analysis consists in recording the optical spectrum of the laser pulse

$$\tilde{E}(\omega) = \mathcal{T}\tilde{E}_0(\omega) + \int_{-\infty}^{+\infty} \mathcal{T} m e^{i\psi} X_{in}(t) E_0(t) e^{-i\omega t} dt, \tag{62}$$

that can be written as a convolution:

$$\tilde{E}(\omega) = \mathcal{T}\tilde{E}_0(\omega) + \mathcal{T}\frac{m e^{i\psi}}{2\pi}\int_{-\infty}^{+\infty} \tilde{X}_{in}(\omega')\tilde{E}_0(\omega - \omega')d\omega', \tag{63}$$

where $\tilde{X}_{in}(\omega)$ is the Fourier transform of the input signal $X_{in}(t)$:

$$\tilde{X}_{in}(\omega) = a\pi e^{i\phi\omega}\left[\delta\left(\omega - \Omega\right) + \delta\left(\omega + \Omega\right)\right]. \tag{64}$$

Using Eq. (7) with $A(-\omega/C)$ slowly varying, Eq. (63) becomes:

$$\tilde{E}(\omega) \approx \mathcal{T}\tilde{E}_0(\omega) + \mathcal{T}\frac{m e^{i\psi}}{2\pi}\sqrt{-\frac{i2\pi}{C}} A\left(-\frac{\omega}{C}\right)\int_{-\infty}^{+\infty} \tilde{X}_{in}(\omega') e^{i\frac{(\omega-\omega')^2}{2C}} d\omega'. \tag{65}$$

Inserting Eq. (64) in Eq. (65):

$$\tilde{E}(\omega) \approx \mathcal{T}\tilde{E}_0(\omega) + \mathcal{T}\sqrt{-\frac{i2\pi}{C}} A\left(-\frac{\omega}{C}\right) e^{i\frac{\omega^2}{2C}} m e^{i\psi} e^{i\frac{\Omega^2}{2C}} a\cos\left(\phi - \frac{\omega}{C}\Omega\right). \tag{66}$$

Hence, the modulated laser spectrum is:

$$\tilde{E}(\omega) = \mathcal{T}\tilde{E}_0(\omega)\left[1 + m e^{i\psi} e^{i\frac{\Omega^2}{2C}} X_{in}\left(-\frac{\omega}{C}\right)\right]. \tag{67}$$

The recorded data consist of the optical spectrum $|\tilde{E}(\omega)|^2$. As we are interested by small signal response, the optical spectrum becomes:

$$|\tilde{E}(\omega)|^2 = |\mathcal{T}\tilde{E}_0(\omega)|^2\left[1 + 2m\cos\left(\psi + \frac{\Omega^2}{2C}\right) X_{in}\left(-\frac{\omega}{C}\right)\right] + O(m^2). \tag{68}$$

We define the output signal, i.e. the measured electro-optic signal, as

$$Y(t) \equiv \frac{|\tilde{E}(\omega)|^2 - |\mathcal{T}\tilde{E}_0(\omega)|^2}{|\mathcal{T}\tilde{E}_0(\omega)|^2}, \tag{69}$$

with the input time coordinate t related to the output spectral coordinate ω by Eq. (9). In case of small input signal, Eq. (69) is equal to:

$$Y(t) \approx 2m\cos\left(\psi + \frac{\Omega^2}{2C}\right) X_{in}\left(t\right). \tag{70}$$

The transfer function $H(\Omega)$ is defined as the ratio between the output and input signal, thus:

$$H(\Omega) = 2m\cos\left(\psi + \frac{\Omega^2}{2C}\right). \tag{71}$$

VIII. NUMERICAL ESTIMATION OF DEOS TEMPORAL RESOLUTION

Note that all durations are FWHM in the following. The reconstruction method is presented in the article (see Materials and Methods) and will not be recalled here. We focus here on the estimation of the resolution of the DEOS method, that is summarized in Figure 1d and the Discussion Section of the Article.

The temporal resolution $\tau_R^{classic}$ of classic spectral encoding has been defined by the shortest Gaussian input THz pulse that can be retrieved without important deformation [4]:

$$\tau_R^{classic} = \sqrt{\tau_L \tau_w}, \tag{72}$$

with τ_w the input chirped laser pulse duration, and τ_L the compressed pulse duration. In order to make a relevant comparison, we estimate here the resolution limit of DEOS in the same conditions, i.e., a crystal with instantaneous response, and noiseless data.

A. Simulation parameters

The simulation parameters given in Table I. The THz pulse shape is a Gaussian centered on the laser pulse.

EO crystal	
n_0	3.1
r_{41}	0.97 pmV^{-1}
d	5 mm
Laser parameters	
λ_0	1040 nm
$\Delta\lambda$	40 nm
τ_L	39 fs FWHM
τ_w	0.1–10 ps FWHM
THz parameters	
E_{THz}	1 kVm^{-1}
τ_{THz}	0.01–1 ps FWHM

TABLE I. Numerical simulation parameters

B. Quality of DEOS measurements for THz input pulses with duration close to τ_L

Before defining a relevant time resolution, it is worth examining the quality of the measurement, when the input duration τ_{THz} becomes close to the compressed laser duration τ_L, and for measurement windows τ_w for which the classical spectral decoding method fails.

We can see that the shape of retrieved input is relatively regular (i.e., remains a bell-like shape) either when:

- $\tau_{THz} = \tau_L$ and τ_w is increased (Figure S7)

- and – at fixed window duration τ_w – when the input time duration τ_{THz} is decreased down to τ_L (Figure S8.

Of course, as expected, the retrieved pulse shape noticeably departs from the input one when the input duration τ_{THz} approaches τ_L. We use this as the basis of the definition for the time resolution of DEOS in this paper.

C. Time resolution of DEOS and comparison to the classical limit $\tau_R^{classic}$

The previous simulations suggest to define the time resolution limit τ_R^{DEOS} of DEOS as:

$$\tau_R^{DEOS} \equiv \tau_{THz}^{retrieved} \text{ when } \tau_{THz} = \tau_L. \tag{73}$$

A typical example is given in Figure S9. Figure S9a shows the retrieved duration $\tau_{THz}^{retrieved}$ versus the input duration τ_{THz}. Note that in this representation, a "perfect" measurement system would provide a response that is exactly on the 45 degree line. Here we can also see that DEOS provides a near-perfect measurement provided the input THz pulse duration τ_{THz} is just a little larger than the laser pulse duration τ_L. The value at $\tau_{THz} = \tau_L$ then provides the temporal resolution τ_R^{DEOS} as defined in Eq. (73).

The resolution of DEOS is plotted in Figure S9b, which corresponds to Figure 1d of the article. This shows that the resolution τ_R^{DEOS} is only slightly larger than the laser pulse duration τ_L, which is in agreement with the intuitive conjecture that $\tau_R^{DEOS} = O(\tau_L)$. However as the most important point, the time resolution does not degrade when the time window τ_w is increased. This represents the main advantage with respect to classical spectraly decoded EO sampling (dashed line).

[1] Casalbuoni, S. *et al.* Numerical studies on the electro-optic detection of femtosecond electron bunches. *Phys. Rev. ST Accel. Beams* **11**, 072802 (2008).

[2] Jiang, Z. & Zhang, X.-C. Electro-optic measurement of

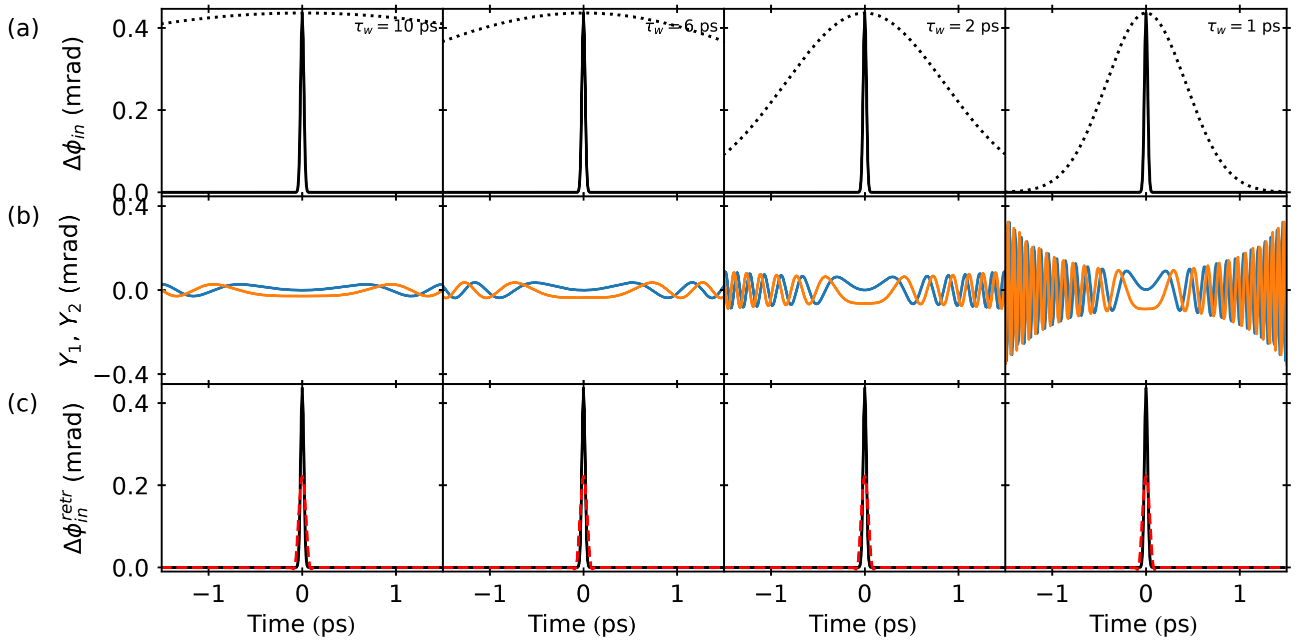

FIG. S7. Numerical simulations of the retrieved signal when a Gaussian THz pulse is applied at the input. (a) Input THz signals (solid line) and chirped probe laser pulse shapes (dotted line). (b) Electro-optic signals Y_1 and Y_2 before DEOS reconstruction. (c) Reconstructed THz input signal (red), and actual THz input signal (black). The input THz signal duration is chosen equal to the compressed laser duration: $\tau_{THz} = \tau_L = 39$ fs FWHM. Other parameters are given in Table I.

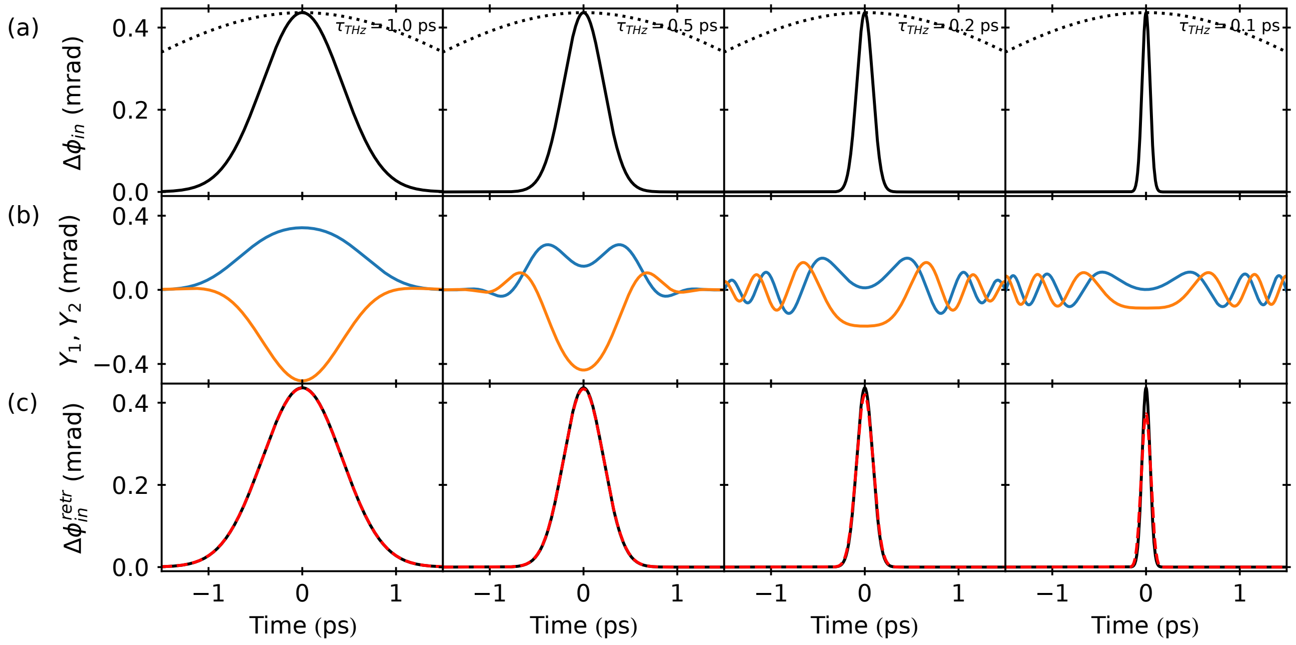

FIG. S8. Same simulations as in Figure S7, for various THz input pulse durations τ_{THz}. (a) Input THz signals (solid line) and chirped probe laser pulse shapes (dotted line). (b) Electro-optic signals Y_1 and Y_2 before DEOS reconstruction. (c) Reconstructed THz input signal (red), and actual THz input signal (black). The chirped laser duration, which defines the acquisition time window, is equal to $\tau_w = 5$ ps FWHM. Other parameters are given in Table I.

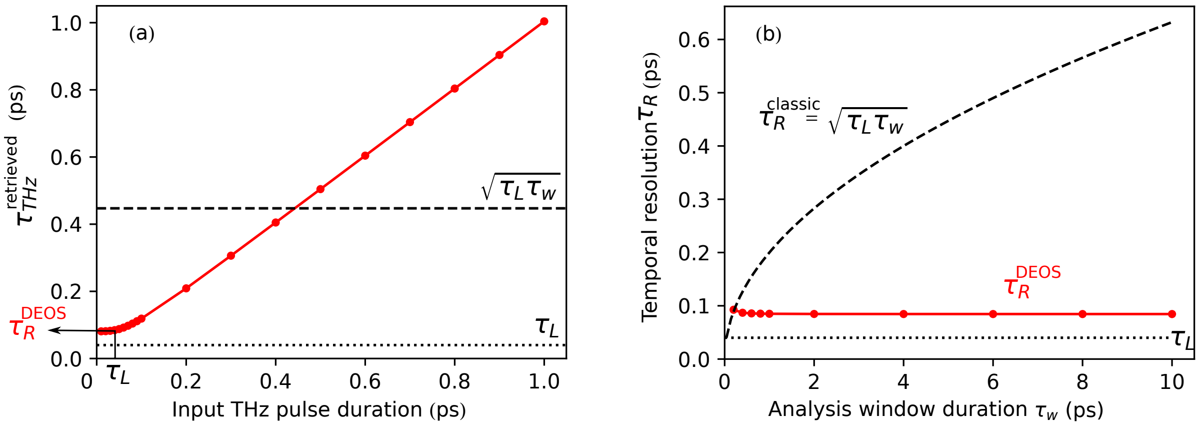

FIG. S9. Numerical study of the time resolution limit of DEOS. (a) Duration $\tau_{THz}^{retrieved}$ of the retrieved input pulse versus the actual duration of the input pulse τ_{THz}. (b) Time resolution τ_R^{DEOS} versus analysis window duration τ_w. The dashed line indicates the time resolution of classical spectrally-decoded EO detection given by Ref. [4]. The dotted line indicates the compressed laser pulse duration τ_L. (a) $\tau_w = 5$ ps FWHM. (a) and (b): $\tau_L = 39\ fs$ FWHM.

THz field pulses with a chirped optical beam. *Appl. Phys. Lett.* **72**, 1945 (1998).

[3] Han, Y., Boyraz, O. & Jalali, B. Ultrawide-band photonic time-stretch A/D converter employing phase diversity. *IEEE Trans. on Microwave Theory and Techniques* **53**, 1404 (2005).

[4] Sun, F., Jiang, Z. & Zhang, X.-C. Analysis of terahertz pulse measurement with a chirped probe beam. *Applied Physics Letters* **73**, 2233–2235 (1998).
